# Prognostic value of stress hyperglycemia ratio, hemoglobin glycation index, and glycemic variability for postoperative atrial fibrillation: a machine learning-based prediction model

**DOI:** 10.1186/s12911-026-03507-z

**Published:** 2026-04-24

**Authors:** Runjia Liu, Chenglong Yao, Hongfan Qiu, Jiatong Li, Ling Yao, Dongdong Su, Haixia Li

**Affiliations:** 1 Department of Cardiology, Guang’anmen Hospital, China Academy of Chinese Medical Sciences, Beijing, China; 2 Shandong University of Traditional Chinese Medicine, Jinan, China; 3 The Second Clinical Medical College of Henan University of Chinese Medicine, Zhengzhou, China

**Keywords:** Stress hyperglycemia ratio, Hemoglobin glycation index, Glycemic variability, Postoperative atrial fibrillation, Machine learning

## Abstract

**Background:**

Postoperative atrial fibrillation (POAF) is a frequent complication following cardiac surgery, linked to higher risks of death, stroke, and extended hospital stays. Given that glycemic control metrics—such as stress hyperglycemia ratio (SHR), glycemic variability (GV), and hemoglobin glycation index (HGI)—have been linked to adverse cardiovascular outcomes, it is important to understand their role in POAF. However, their comparative predictive value for POAF remains unclear. Therefore, this study aimed to evaluate the predictive value of SHR, GV, and HGI for POAF and to develop a machine learning-based model for POAF risk prediction.

**Methods:**

We retrospectively analyzed 2,177 cardiac surgery patients from the MIMIC-IV database (median age 69 years [IQR 60–76]; 69.4% male). SHR, GV, and HGI were calculated from postoperative glucose measurements. Associations with POAF were examined using multivariable logistic regression, restricted cubic splines, threshold effect, and subgroup analyses. Thirteen machine learning algorithms were compared to develop a prediction model, with variable importance assessed using SHapley Additive exPlanations (SHAP). The final model was implemented as an interactive Shiny web application.

**Results:**

POAF incidence was 39.8%. SHR and HGI were identified as independent predictors (SHR: OR = 1.39, 95% CI 1.01–1.93, *P* = 0.049; HGI: OR = 0.90, 95% CI 0.82–0.98, *P* = 0.017). Restricted cubic spline analysis demonstrated a negative linear association between HGI and POAF risk (P for overall = 0.039; P for nonlinearity = 0.375). In contrast, SHR showed a nonlinear relationship with POAF, with risk increasing above an inflection point of 0.9067 (P for overall = 0.019; P for nonlinearity = 0.042). GV also exhibited a significant nonlinear association with POAF risk (*P* = 0.025; P for nonlinearity = 0.049). The final AdaBoost model achieved an AUC of 0.74 (95% CI% 0.684–0.778), demonstrating moderate discrimination. In contrast, the SHR-only model yielded an AUC of 0.557 (95% CI% 0.512–0.602). These findings indicate that integrating multidimensional clinical variables substantially improves predictive performance compared with a single metabolic indicator. However, as the model was developed using a single database with internal validation only, external validation in independent cohorts is required before clinical implementation.

**Conclusion:**

SHR and HGI are independent predictors of POAF, and GV shows a threshold effect. An AdaBoost-based web tool enables individualized, rapid perioperative POAF risk estimation, potentially supporting tailored prevention strategies.

**Graphical Abstract:**

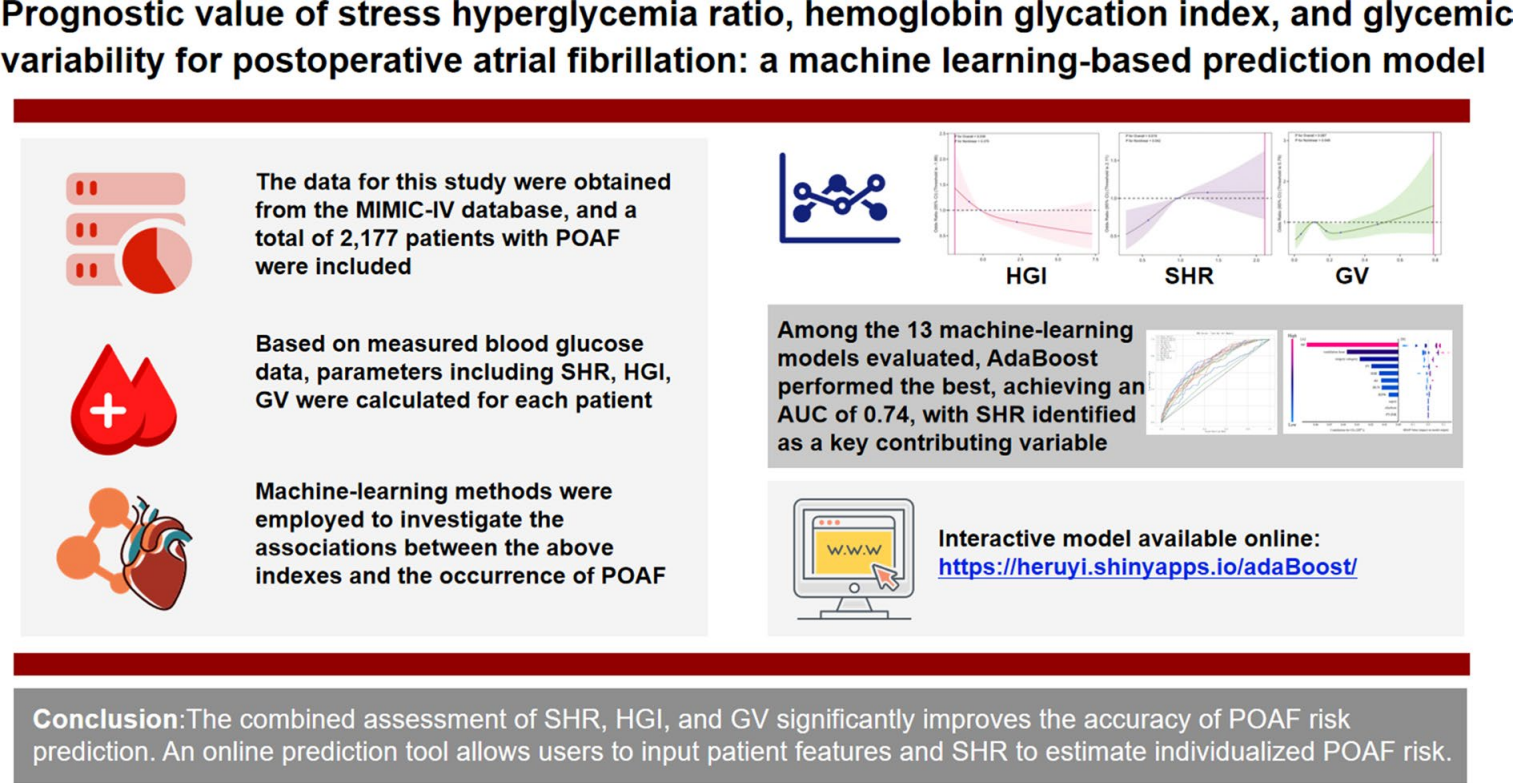

**Supplementary Information:**

The online version contains supplementary material available at 10.1186/s12911-026-03507-z.

## Introduction

Postoperative atrial fibrillation (POAF) is one of the most common and serious complications of cardiac surgery, with an incidence rate of 15% to 40% [1]. POAF not only significantly prolongs patients’ hospital stay and medical costs [2, 3], but is also closely associated with an increased risk of stroke, heart failure, and death [3–5]. Although multiple mechanisms such as postoperative inflammatory response, oxidative stress, and autonomic dysfunction are believed to be involved in the occurrence of POAF, its specific metabolic risk factors have not yet been fully clarified, and more accurate risk assessment tools are urgently needed to optimize perioperative management.

Abnormal glucose metabolism is closely related to cardiovascular disease. Previous studies have shown that acute glycemic stress, blood glucose fluctuations, and chronic glucose metabolism abnormalities may affect cardiac electrophysiological stability and inflammatory response. The stress hyperglycemia ratio (SHR) is an indicator that measures acute stress hyperglycemia relative to chronic blood glucose control. It was first proposed by Roberts et al. to more accurately assess the severity of hyperglycemia under stress [6]. Glycemic variability (GV) reflects the amplitude of blood glucose fluctuations in the short term. The hemoglobin glycation index (HGI) reflects the differences in individual long-term blood glucose regulation and reveals the deviation between HbA1c and actual blood glucose levels. It was proposed by Hempe et al. [7]. As a supplementary indicator of chronic glycemic load, its potential value in cardiovascular risk prediction is increasingly attracting attention. However, there is currently a lack of systematic research on the joint analysis of multidimensional blood glucose metabolism indicators in postoperative arrhythmia, especially the construction and verification of risk models based on big data.

Notably, these three metrics capture distinct but complementary dimensions of glucose metabolism. SHR reflects acute stress-related hyperglycemia relative to baseline glycemic status, GV quantifies short-term glucose fluctuations that may directly affect electrophysiological stability, and HGI represents interindividual differences in long-term glycation independent of measured glucose levels. Together, they provide a multidimensional characterization of acute, dynamic, and chronic glycemic burden. Compared with traditional indicators such as fasting glucose or HbA1c alone, these composite metrics may better capture perioperative metabolic stress and its arrhythmogenic potential.

Previous studies have shown that SHR, GV, and HGI are all associated with adverse cardiovascular outcomes [8–12]. Given the multifactorial pathogenesis of POAF, the predictive ability of a single indicator is limited. Comprehensive evaluation of multidimensional blood glucose indicators may improve the accuracy and clinical value of risk prediction. In addition, with the development of artificial intelligence and machine learning technologies [13], they are also widely used in clinical medicine [14]. Prediction models based on machine learning can handle high-dimensional complex variables, optimize risk stratification, and improve prediction performance [15].

To date, no study has simultaneously evaluated SHR, HGI, and GV for POAF, nor has any machine learning model examined the impact of these three metrics on POAF.

Therefore, we hypothesized that integrating acute (SHR), dynamic (GV), and chronic deviation (HGI) glycemic metrics may provide superior predictive value for POAF compared with single indicators. This study aimed to [1] systematically evaluate the independent and nonlinear associations of SHR, GV, and HGI with POAF [2], compare their relative predictive performance, and [3] develop and validate a machine learning–based model incorporating these multidimensional glycemic markers to improve individualized POAF risk stratification.

## Methods

This retrospective cohort study analyzed 2,177 cardiac surgery patients admitted to the ICU using the MIMIC-IV 3.1 database. To investigate the relationship between three glycemic control indicators and the risk of POAF, patients were categorized into tertiles for SHR, HGI, and GV. A multivariate logistic regression model was used to assess independent associations after adjusting for confounding factors such as demographic characteristics, vital signs, underlying medical conditions, severity scores, and surgical type. Restricted cubic splines (RCS) were used to characterize the nonlinear relationship between glycemic indicators and POAF risk. Sensitivity and subgroup analyses were also performed to validate the findings.

To assess the importance of variables in predicting POAF, the Boruta feature selection algorithm was used to identify the most predictive features from clinical variables. Furthermore, a postoperative AF prediction model was constructed using SHR as the core variable. During model training, 13 different machine learning algorithms (including AdaBoost, RandomForest, DecisionTree, SVM, LDA, Ridge, LogReg, KNN, Naive Bayes, GBM, NN, MLP, and XGBoost ) were applied, and model performance was evaluated using five-fold cross-validation. Area under the curve (AUC) was the primary evaluation metric, with accuracy, precision, recall, and F1 score also reported. Model calibration was evaluated using calibration plots and the Brier score.

To enhance the clinical interpretability of the model, the SHAP algorithm was used to interpret the final model and demonstrate the marginal contribution of each variable to the individual predicted risk. Furthermore, the model performance was validated using DCA.

### Study population

This retrospective cohort study used data from the MIMIC-IV database (v3.1) [16], a large public repository of de-identified records from patients admitted to intensive care units at Beth Israel Deaconess Medical Center from 2008 to 2022. Access was granted through the PhysioNet platform after one author completed the CITI program, passing the “Conflict of Interest” and “Research with Data or Samples Only” modules (Certification ID: 13975929).

This study followed the Declaration of Helsinki principles and adhered to the Strengthening the Reporting of Observational Studies in Epidemiology (STROBE) guidelines [17]. Informed consent was waived as only de-identified data were used.

In the MIMIC-IV, all patients undergoing coronary artery bypass grafting(CABG), heart valve surgery, or aortic surgery were eligible for inclusion.

### Data extraction and definitions

All participants in this study had undergone CABG, valve surgery, or aortic surgery and were subsequently admitted to the intensive care unit (ICU). Cardiac surgery cases were identified using ICD-9 and ICD-10 procedure codes, with detailed diagnoses listed in Supplementary Table S1. Patients were further excluded according to the following criteria:

1. Age < 18 years old (*n* = 0).
2. ICU stay of less than 1 day (*n* = 556).
3. Patients admitted to the ICU after non-cardiac surgery (*n* = 263).
4. Patients lacked basic data such as blood glucose or HbA1c on the first day of ICU admission.(*n* = 4704).
5. Patients with a history of AF or preoperative AF (*n* = 1154).
6. Patients who measured blood glucose less than twice (*n* = 934).

A total of 2177 eligible patients were selected and divided into three groups based on their SHR, HGI, and GV values.

**Fig. 1:**
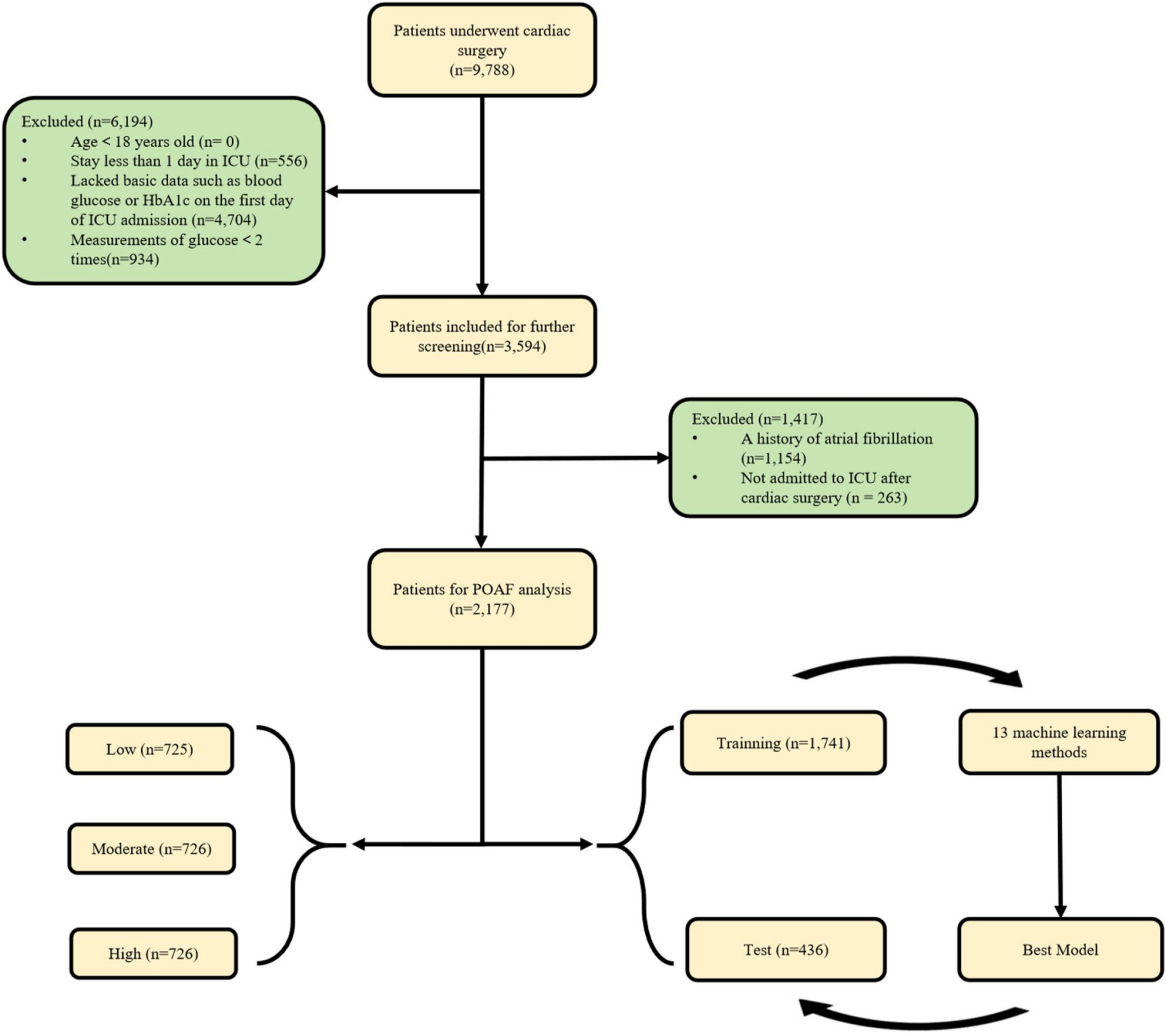
Flow chart

The analysis was limited to those admitted to the ICU after cardiac surgery. A flowchart of the patient enrollment process is shown in Fig. 1.

#### POAF, postoperative atrial fibrillation

Demographic information, vital signs, ICU medications and treatments, comorbidities, surgical details, and laboratory results were retrieved using Navicat Premium (version 16.3.2) with structured query language (SQL).

The extracted data included baseline characteristics :

1. Demographic data, including age, sex, race, BMI, and marital status at admission.
2. Comorbidities, including hypertension, acute kidney injury(AKI), myocardial infarction, liver cirrhosis, pneumonia, cerebrovascular accident, chronic kidney disease, cancer, chronic obstructive pulmonary disease (COPD), diabetes, heart failure, ischemic heart disease, and POAF.
3. Scores: Charlson Comorbidity Index (CCI), Sequential Organ Failure Assessment (SOFA), Acute Physiology Score III (APSIII), Simplified Acute Physiology Score II (SAPS-II), Oxford Acute Severity of Disease Score (OASIS), Systemic Inflammatory Response Syndrome (SIRS), and Glasgow Coma Scale (GCS).
4. Vital signs, systolic blood pressure (SBP), diastolic blood pressure (DBP), mean blood pressure (MBP), arterial blood pressure systolic (ABPs), arterial blood pressure diastolic (ABPd), mean arterial blood pressure (ABPm), pulse oxygen saturation (SpO₂), body temperature (Temperature Fahrenheit, Temperature F).
5. Laboratory tests: Hemoglobin (Hb), hematocrit (HCT), red blood cells (RBC), white blood cells (WBC), platelet count (PLT), red cell distribution width (RDW), neutrophil count (ANC), lymphocyte count (ALC), albumin (ALB), globulin (GLB), total protein (TP), urea nitrogen (BUN), uric acid (UA), alanine aminotransferase (ALT), aspartate aminotransferase(AST), lactate dehydrogenase (LDH), total bilirubin (TBIL), direct bilirubin (DBIL), indirect bilirubin (IBIL), creatine kinase (CK), creatine kinase MB isoenzyme (CK-MB), N-terminal pro-B-type natriuretic peptide (NTproBNP), troponin I (TnI), troponin T (TnT), fibrinogen functional (FIB), D-dimer (DD), prothrombin time (PT), partial thromboplastin time (PTT), international normalized ratio (INR), thrombin (THR), total cholesterol (TC), triglycerides (TG), high-density lipoprotein cholesterol (HDL-C), and low-density lipoprotein cholesterol (LDL-C).
6. Treatment, including the use of CRRT (Continuous renal replacement therapy, CRRT), mechanical ventilation and ventilation hour, and whether medications were used: statins, amiodarone, magnesium, vasopressors, glucocorticoids, beta-blockers, and insulin.

The above values were recorded as the first measurement starting from the first day of ICU admission.

The medication extraction time is 7 days before surgery.

### Definition of POAF

This study defined POAF as the first onset of atrial fibrillation during hospitalization after cardiac surgery. To ensure that only cases with new-onset AF were included, we excluded all patients with a preoperative history of atrial fibrillation, including any reference to prior atrial fibrillation in diagnostic codes, continuous electrocardiogram (ECG) monitoring reports, or medical records. Only the first postoperative episode of atrial fibrillation was included.

POAF was identified based on ECG diagnosis, monitoring records, and physician-written medical records. All episodes were included in the analysis, regardless of whether they were persistent or transient [18, 19].

### Definitions of SHR, HGI, and GV

SHR was calculated as:

$${\rm{SHR = }}{\matrix{{\rm{first}}\,{\rm{postoperative}}\> \hfill \cr {\rm{blood}}\>{\rm{glucose}}\>{\rm{(mg/dL)}} \hfill \cr} \over {{\rm{28}}.{\rm{7}}\> \times \>{\rm{HbA1c}}\>(\% )\> - \>{\rm{46}}.{\rm{7}}}}$$

where the first postoperative blood glucose refers to the first glucose measurement obtained after surgery upon ICU admission.

HGI was calculated as:

$$\eqalign{{\rm{HGI = }} & {\rm{HbA1c}} \cr & {\rm{ - }}({\rm{0}}.{\rm{009}} \times {\rm{first}}{\mkern 1mu} {\rm{glucose}}{\mkern 1mu} {\rm{measurement + 4}}.{\rm{940}}), \cr} $$

where the first glucose measurement refers to the initial glucose value recorded after ICU admission.

All postoperative blood glucose measurements were used to calculate the coefficient of variation (CV), defined as the ratio of standard deviation to mean glucose level.

### Study endpoint

The primary endpoint of this study was the incidence of postoperative atrial fibrillation (POAF) during hospitalization.

All association analyses, non-linear modeling, and machine learning prediction models were performed using POAF as the outcome variable.

### Statistical analysis

Variables with > 15% missing data were excluded, while those with ≤ 15% missing data were imputed using the random forest method using the random forest method. Patients were stratified according to tertiles of SHR, HGI, andGV. These tertiles were designated as T1 (low group), T2 (intermediate group), and T3 (high group). Specific tertile cutoffs were as follows: <0.85 (T1), 0.85–1.08 (T2), and > 1.08 (T3) for SHR; <0.13 (T1), 0.13–0.24 (T2), and > 0.24 (T3) for GV; and <-0.46 (T1), -0.46 to -0.00 (T2), and > 0.28 (T3) for HGI.

Continuous variables were presented as mean ± SD or median (IQR) and compared using Student’s t-test or Kruskal-Wallis test, as appropriate. Categorical data were shown as counts (percentages) and analyzed using chi-square or Fisher’s exact test.

To minimize potential confounding, multivariable logistic regression was applied to evaluate the independent relationships of SHR, HGI, and GV with the occurrence of POAF. Candidate covariates were determined according to both clinical relevance and statistical significance identified in univariate analyses (*p* < 0.05). Prior to entering the final model, multicollinearity among these variables was examined using the variance inflation factor (VIF). Covariates with a VIF > 10 were removed to ensure model stability.

Furthermore, a RCS model was used to analyze the nonlinear dose-effect relationship between SHR, HGI, and GV and the risk of POAF. Stratified analyses were performed to explore the consistency of the predictive value of each indicator for POAF across different subgroups, with forest plots presenting the ORs and 95 confidence intervals for each subgroup. Subgroup analyses were performed according to age, sex, and comorbid conditions to determine whether the associations of SHR, HGI, and GV with the primary endpoint were consistent across diverse patient strata.

In addition, the Boruta feature selection algorithm was used to identify important variables associated with POAF. Machine learning models, including AdaBoost, RandomForest, DecisionTree, SVM, LDA, Ridge, LogReg, KNN, Naive Bayes, GBM, NN, MLP, and XGBoost, were constructed to predict the occurrence of postoperative AF. The dataset was randomly split into training and testing sets at a ratio of 8:2. Model performance was compared using AUC, accuracy, F1 value, sensitivity, and specificity. The SHAP algorithm was employed to interpret the predictions of the optimal model and to determine the most influential variables associated with POAF. Statistical analyses were conducted using Python (v3.11), R (v4.4.3), and DecisionLnnc (v3.10.6). Statistical significance was set at a two-tailed p value < 0.05.

## Results

### Baseline characteristics

A total of 2,177 patients undergoing cardiac surgery and admitted to the ICU were included in this study. The median age of the overall cohort was 69 years (IQR 60–76), and 69.4% were male. Among them, 867 patients (39.8%) developed POAF during hospitalization. Patients were subsequently divided into a POAF group (*n* = 867) and a non-POAF group (*n* = 1,310) for comparative analysis of baseline characteristics (Table 1).

Compared with patients without POAF, patients in the POAF group were significantly older (median 72 vs. 66 years, *P* < 0.001) and had a significantly higher incidence of underlying medical conditions, including cerebrovascular accident, acute kidney injury, malignancy, and heart failure (*P* < 0.05).

Regarding vital signs, diastolic blood pressure was slightly lower in the POAF group (*P* = 0.006), and SOFA, APSIII, SAPSII, OASIS, and CCI were significantly higher in the POAF group than in the non-POAF group (all *P* < 0.001), suggesting that their overall condition was more severe.

Regarding laboratory test results, patients in the POAF group exhibited markedly higher BUN, INR, PT, and RDW values, whereas hemoglobin and hematocrit were comparatively reduced (*p* < 0.05). In addition, the SHR was substantially greater in the POAF cohort than in the control cohort, while the HGI was significantly lower in the POAF group (both *p* < 0.001). No meaningful difference in GV was observed between the two groups.

For surgical procedure type, patients who underwent combined CABG and valve surgery (CABG + Valve) exhibited a significantly higher incidence of POAF (*P* < 0.001), indicating that combined procedures may elevate POAF risk. Additionally, the POAF group showed a higher frequency of continuous renal replacement therapy (CRRT) utilization (*P* = 0.003).

**Table 1:** Baseline characteristics of patients stratified by POAF occurrence

Variable	Levels	Overall (*N* = 2,177)	Without POAF (*N* = 1,310)	With POAF (*N* = 867)	*p*-value
**Demographic Characteristics**					
Age, years		69.00 (60.00–76.00)	66.00 (57.00–74.00)	72.00 (65.00–78.00)	< 0.001
Gender, n (%)					0.721
	Female	666.00 (30.59)	397.00 (30.31)	269.00 (31.03)	
	Male	1,511.00 (69.41)	913.00 (69.69)	598.00 (68.97)	
Race, n (p)					< 0.001
	Black	111.00 (5.10)	79.00 (6.03)	32.00 (3.69)	
	White	1,434.00 (65.87)	812.00 (61.98)	622.00 (71.74)	
	Other	632.00 (29.03)	419.00 (31.98)	213.00 (24.57)	
BMI, kg/m2		28.91 (25.66–33.23)	28.87 (25.59–32.85)	28.96 (25.68–33.56)	0.192
Marital status, n (p)					< 0.001
	Divorced	149.00 (6.84)	93.00 (7.10)	56.00 (6.46)	
	Married	1,399.00 (64.26)	828.00 (63.21)	571.00 (65.86)	
	Single	427.00 (19.61)	288.00 (21.98)	139.00 (16.03)	
	Widowed	202.00 (9.28)	101.00 (7.71)	101.00 (11.65)	
**Vital Signs**					
SpO_2,_		100.00 (99.00–100.00)	100.00 (99.00–100.00)	100.00 (99.00–100.00)	0.076
Temperature, F		97.90 (97.60–98.40)	97.90 (97.60–98.40)	97.95 (97.60–98.40)	0.018
Heart Rate, beats/min		80.00 (76.00–85.00)	80.00 (76.00–85.08)	80.00 (75.00–85.00)	0.403
Systolic blood pressure, mmHg		108.62 (99.00–120.00)	108.00 (99.00–119.00)	109.00 (99.00–121.00)	0.287
Diastolic blood pressure, mmHg		60.00 (54.00–67.00)	60.64 (54.00–68.00)	58.72 (52.00–66.00)	0.006
Mean arterial pressure, mmHg		72.33 (67.00–80.00)	73.00 (67.00–80.14)	72.00 (66.00–80.00)	0.181
Respiratory Rate, times/min		15.06 (13.00–17.00)	15.00 (13.00–17.00)	15.28 (13.00–17.10)	0.998
**Comorbidities**					
Hypertension, n (p)	Yes	1,070.00 (49.15)	666.00 (50.84)	404.00 (46.60)	0.053
Acute Kidney Injury, n (p)	Yes	529.00 (24.30)	253.00 (19.31)	276.00 (31.83)	< 0.001
Liver Cirrhosis, n (p)	Yes	25.00 (1.15)	14.00 (1.07)	11.00 (1.27)	0.668
Pneumonia, n (p)	Yes	165.00 (7.58)	91.00 (6.95)	74.00 (8.54)	0.170
Cerebrovascular Accident, n (p)	Yes	184.00 (8.45)	96.00 (7.33)	88.00 (10.15)	0.021
Chronic Kidney Disease, n (p)	Yes	434.00 (19.94)	233.00 (17.79)	201.00 (23.18)	0.002
Cancer, n (p)	Yes	286.00 (13.14)	148.00 (11.30)	138.00 (15.92)	0.002
Diabetes Mellitus, n (p)	Yes	854.00 (39.23)	527.00 (40.23)	327.00 (37.72)	0.240
Hyperlipidemia, n (p)	Yes	1,469.00 (67.48)	884.00 (67.48)	585.00 (67.47)	0.997
Heart Failure, n (p)	Yes	757.00 (34.77)	402.00 (30.69)	355.00 (40.95)	< 0.001
Myocardial Infarction, n (p)	Yes	502.00 (23.06)	310.00 (23.66)	192.00 (22.15)	0.410
Ischemic Heart Disease, n (p)	Yes	1,750.00 (80.39)	1,063.00 (81.15)	687.00 (79.24)	0.273
COPD, n (p)	Yes	228.00 (10.47)	124.00 (9.47)	104.00 (12.00)	0.059
**Score**					
SOFA		5.00 (3.00–7.00)	5.00 (3.00–7.00)	5.00 (4.00–8.00)	< 0.001
APSIII		36.00 (28.00–46.00)	34.00 (27.00–44.00)	37.00 (29.00–48.00)	< 0.001
SAPSII		35.00 (29.00–43.00)	34.00 (27.00–41.00)	37.00 (31.00–45.00)	< 0.001
OASIS		30.00 (25.00–35.00)	30.00 (24.00–35.00)	31.00 (26.00–37.00)	< 0.001
GCS		15.00 (14.00–15.00)	15.00 (14.00–15.00)	15.00 (14.00–15.00)	0.126
CCI		4.00 (3.00–6.00)	4.00 (3.00–6.00)	5.00 (3.00–7.00)	< 0.001
SIRS, n (p)					0.691
	0	6.00 (0.28)	5.00 (0.38)	1.00 (0.12)	
	1	155.00 (7.12)	94.00 (7.18)	61.00 (7.04)	
	2	612.00 (28.11)	360.00 (27.48)	252.00 (29.07)	
	3	1,022.00 (46.95)	624.00 (47.63)	398.00 (45.91)	
	4	382.00 (17.55)	227.00 (17.33)	155.00 (17.88)	
**Laboratory indicators**					
Glucose, mg/ dL		119.00 (105.00–138.00)	119.00 (104.00–137.00)	120.00 (106.00–138.00)	0.092
Anion Gap, mEq/L		11.00 (9.00–13.00)	11.00 (9.00–13.00)	11.00 (9.00–13.00)	0.237
Calcium, mg/dl		8.20 (7.90–8.50)	8.20 (7.90–8.50)	8.20 (8.00–8.50)	0.149
Creatinine, mg/dl		0.90 (0.70–1.10)	0.90 (0.70–1.10)	0.90 (0.70–1.20)	0.059
Potassium, mmol/ L		4.40 (4.00–4.70)	4.40 (4.00–4.70)	4.30 (4.00–4.70)	0.867
Sodium, mEq/L		138.00 (136.00–140.00)	138.00 (136.00–140.00)	138.00 (136.00–140.00)	0.160
BUN, mg/dl		16.00 (12.00–21.00)	15.00 (12.00–20.00)	17.00 (13.00–23.00)	0.001
Fibrinogen Functional, mg/dL		229.00 (188.00–276.00)	229.00 (189.00–273.00)	228.00 (186.00–280.00)	0.986
Hematocrit,		27.50 (24.40–31.00)	27.80 (24.60–31.20)	27.20 (24.10–30.80)	0.031
Hemoglobin, g /L		9.10 (7.90–10.40)	9.20 (8.00–10.50)	8.90 (7.80–10.20)	0.002
INRPT		1.40 (1.30–1.60)	1.40 (1.30–1.60)	1.50 (1.30–1.70)	< 0.001
PLT, K/uL		142.00 (113.00–178.00)	144.00 (114.00–183.00)	138.00 (110.00–172.00)	0.001
PT, s		15.80 (14.40–17.50)	15.50 (14.20–17.10)	16.25 (14.80–18.10)	< 0.001
PTT		31.00 (27.40–36.40)	30.40 (27.20–35.50)	31.80 (27.90–37.30)	0.507
RBC Distribution Width,		13.60 (12.90–14.70)	13.50 (12.80–14.60)	13.80 (13.10–14.90)	< 0.001
RBC, K/uL		3.05 (2.65–3.46)	3.07 (2.69–3.49)	3.00 (2.61–3.38)	< 0.001
WBC, K/uL		12.40 (9.10–16.30)	12.40 (9.00–16.40)	12.50 (9.30–16.10)	0.714
Ventilation hour		38.73 (18.52–64.00)	33.35 (16.00–52.50)	45.82 (22.25–76.33)	< 0.001
GV		0.18 (0.11–0.27)	0.18 (0.11–0.27)	0.18 (0.12–0.27)	0.183
HGI		-0.16 (-0.61–0.71)	-0.11 (-0.60–0.84)	-0.23 (-0.63–0.53)	< 0.001
SHR		0.96 (0.77–1.15)	0.95 (0.75–1.14)	0.98 (0.82–1.17)	< 0.001
**Therapy**					
Surgery Category, n (p)					< 0.001
	CABG	1,082.00 (49.70)	724.00 (55.27)	358.00 (41.29)	
	Valve	613.00 (28.16)	330.00 (25.19)	283.00 (32.64)	
	Aorta	7.00 (0.32)	5.00 (0.38)	2.00 (0.23)	
	CABG+Valve	430.00 (19.75)	224.00 (17.10)	206.00 (23.76)	
	CABG+Aortic	3.00 (0.14)	1.00 (0.08)	2.00 (0.23)	
	Valve+Aortic	37.00 (1.70)	23.00 (1.76)	14.00 (1.61)	
	CABG+Valve+Aortic	5.00 (0.23)	3.00 (0.23)	2.00 (0.23)	
CRRT, n (p)	Yes	62.00 (2.85)	26.00 (1.98)	36.00 (4.15)	0.003
ventilation, n (p)	Yes	1,962.00 (90.12)	1,173.00 (89.54)	789.00 (91.00)	0.263
Statin, n (p)	Yes	54.00 (2.48)	32.00 (2.44)	22.00 (2.54)	0.889
Amiodarone, n (p)	Yes	6.00 (0.28)	2.00 (0.15)	4.00 (0.46)	0.179
Mg, n (p)	Yes	15.00 (0.69)	10.00 (0.76)	5.00 (0.58)	0.606
Insulin, n (p)	Yes	19.00 (0.87)	13.00 (0.99)	6.00 (0.69)	0.461
Vasopressor, n (p)	Yes	31.00 (1.42)	20.00 (1.53)	11.00 (1.27)	0.619
Glucocorticoid, n (p)	Yes	43.00 (1.98)	32.00 (2.44)	11.00 (1.27)	0.054
Beta-blocker, n (p)	Yes	47.00 (2.16)	24.00 (1.83)	23.00 (2.65)	0.197

Abbreviations: POAF, postoperative atrial fibrillation; BMI, body mass index; SpO₂, peripheral oxygen saturation; COPD, chronic obstructive pulmonary disease; SOFA, Sequential Organ Failure Assessment; APSIII, Acute Physiology Score III; SAPSII, Simplified Acute Physiology Score II; OASIS, Oxford Acute Severity of Illness Score; GCS, Glasgow Coma Scale; CCI, Charlson Comorbidity Index; SIRS, systemic inflammatory response syndrome; BUN, blood urea nitrogen; INRPT, international normalized ratio of prothrombin time; PLT, platelet count; PT, prothrombin time; PTT, partial thromboplastin time; RBC, red blood cell; WBC, white blood cell; GV, glycemic variability; HGI, hemoglobin glycation index; SHR, stress hyperglycemia ratio; CABG, coronary artery bypass grafting; CRRT, continuous renal replacement therapy

**Table 2:** Relationship between HGI, SHR, GV and POAF

Outcomes exposure	Unjusted OR (95% CI, *P*)	Model 1 OR (95% CI, *P*)	Model 2 OR (95% CI, *P*)	Model 3 OR (95% CI, *P*)	Model 4 OR (95% CI, *P*)	
**SHR**						
T1	Ref	Ref	Ref	Ref	Ref	
T2	1.33 (1.07–1.64, *P* = 0.009 )	1.29 (1.04–1.61, *P* = 0.023)	1.30 (1.04–1.63, *P* = 0.021)	1.25 (0.98–1.60, *P* = 0.075)	1.24 (0.96–1.56, *P* = 0.096)	
T3	1.42 (1.15–1.75, *P* = 0.001 )	1.51 (1.21–1.89, *P* < 0.001)	1.51 (1.21–1.90, *P* < 0.001)	1.35 (1.05–1.75, *P* = 0.020)	1.35 (1.04–1.75, *P* = 0.027)	
P for trend	1.19 (1.07–1.32, *P* = 0.001 )	1.23 (1.10–1.37, *P* < 0.001)	1.23 (1.10–1.38, *P* < 0.001)	1.16 (1.02–1.32, *P* = 0.023)	1.17 (1.02–1.33, *P* = 0.023)	
Continuous	1.62 (1.25–2.12, *P* < 0.001 )	1.65 (1.25–2.19, *P* < 0.001)	1.65 (1.25–2.20, *P* < 0.001)	1.35 (1.01–1.85, *P* = 0.057)	1.39 (1.01–1.93, *P* = 0.049)	
**HGI**						
T1	Ref	Ref	Ref	Ref	Ref	
T2	1.00 (0.81–1.24,*P* = 0.975 )	0.83 (0.66,1.03, *P* = 0.096)	0.83 (0.66–1.03, *P* = 0.095)	0.91 (0.72–1.14, *P* = 0.400)	0.94 (0.74–1.19, *P* = 0.581)	
T3	0.78 (0.63–0.96,***P* = 0.020** )	0.66 (0.53–0.83, *P* < 0.001)	0.65 (0.52–0.82, *P* < 0.001)	0.72 (0.53–0.97, *P* = 0.033)	0.72 (0.52–0.99, *P* = 0.041)	
P for trend	0.88 (0.79–0.98,***P* = 0.020** )	0.82 (0.73–0.91, *P* < 0.001 )	0.81 (0.72–0.91, *P* < 0.001)	0.86 (0.74–0.99, *P* = 0.041)	0.86 (0.74–1.01, *P* = 0.059)	
Continuous	0.88(0.82–0.93, *P* = 0.000 )	0.88 (0.82–0.94, *P* < 0.001)	0.88 (0.82–0.94, *P* < 0.001)	0.90 (0.83–0.98, *P* = 0.021 )	0.90 (0.82–0.98, P **= 0.017**)	
**GV**						
T1	Ref.	Ref.	Ref.	Ref.	Ref.	
T2	1.09 (0.88–1.34,*P* = 0.434 )	1.00 (0.80–1.25,*P* = 0.988)	1.00 (0.80–1.25, *P* = 0.984)	0.91 (0.72–1.14, *P* = 0.419)	0.86 (0.68–1.08, *P* = 0.202)	
T3	1.05 (0.85–1.30,*P* = 0.644 )	0.97 (0.78–1.21, *P* = 0.813)	0.97 (0.78–1.21, *P* = 0.780)	0.95 (0.74–1.22, *P* = 0.700)	0.95 (0.74–1.22, *P* = 0.693)	
P for trend	1.03 (0.92–1.14,*P* = 0.645 )	0.99 (0.88–1.10, *P* = 0.813)	0.98 (0.88–1.10, *P* = 0.780)	0.97 (0.86–1.10, *P* = 0.681)	0.97 (0.86–1.10, *P* = 0.650)	
Continuous	1.52 (0.83–2.79, *P* = 0.177)	1.32 (0.70–2.49, *P* = 0.387)	1.29 (0.68–2.45, *P* = 0.429)	1.42 (0.70–2.88, *P* = 0.337)	1.44 (0.69–2.98, *P* = 0.330)	

Model 1 Age, Gender, Race, BMI

Model 2 + SBP, DBP, Respiratory Rate, SpO_2_, Heart rate, Temperature

Model 3 + Comorbidities (Hypertension, Acute Kidney Injury, Liver Cirrhosis, Pneumonia, Cerebrovascular Accident, Chronic Kidney Disease, Cancer, Diabetes Mellitus, Heart Failure, Myocardial Infarction, Ischemic Heart Disease, COPD), Scores (SOFA), Surgery Category

Model 4 + Medications(Statin, Amiodarone, Mg, Vasopressor, Glucocorticoid, Beta-blocker, Insulin) + Lab Tests (anion gap, potassium, sodium, Calcium, BUN, hematocrit, Hemoglobin, INRPT, PLT, PT, RDW, RBC, ventilation hour, WBC) , CRRT

Abbreviations: POAF, postoperative atrial fibrillation; SHR, stress hyperglycemia ratio; HGI, hemoglobin glycation index; GV, glycemic variability; OR, odds ratio; CI, confidence interval; SBP, systolic blood pressure; DBP, diastolic blood pressure; BMI, body mass index; SpO₂, peripheral oxygen saturation; COPD, chronic obstructive pulmonary disease; SOFA, Sequential Organ Failure Assessment; BUN, blood urea nitrogen; INRPT, international normalized ratio of prothrombin time; PLT, platelet count; PT, prothrombin time; RDW, red blood cell distribution width; RBC, red blood cell count; WBC, white blood cell count; CRRT, continuous renal replacement therapy; CABG, coronary artery bypass grafting

### Association between glycemic indicators and POAF

We constructed four multivariate logistic regression models (Table 2) and performed VIF analysis to exclude collinear variables (Supplementary Table S2) to evaluate the association between SHR, GV, and HGI and the risk of POAF. After comprehensive adjustment for confounding factors (model 4), SHR and HGI remained significant independent predictors of POAF (SHR: OR = 1.39, 95% CI:1.01–1.93, *P* = 0.049; HGI: OR = 0.90, 95% CI:0.82–0.98, *P* = 0.017). The risk of POAF was 34.7% higher in patients with SHR in the high tertile compared with those in the low tertile (OR = 1.35, 95% CI: 1.04–1.75, *P* = 0.027). Patients with a high HGI had an approximately 10% lower risk of POAF compared with those with a low HGI (OR = 0.90, 95% CI: 0.82–0.98, *P* = 0.017). SHR and POAF showed a dose-response relationship (P for trend = *P* = 0.023 in Model 4), while GV did not show statistical significance.

**Fig. 2:**
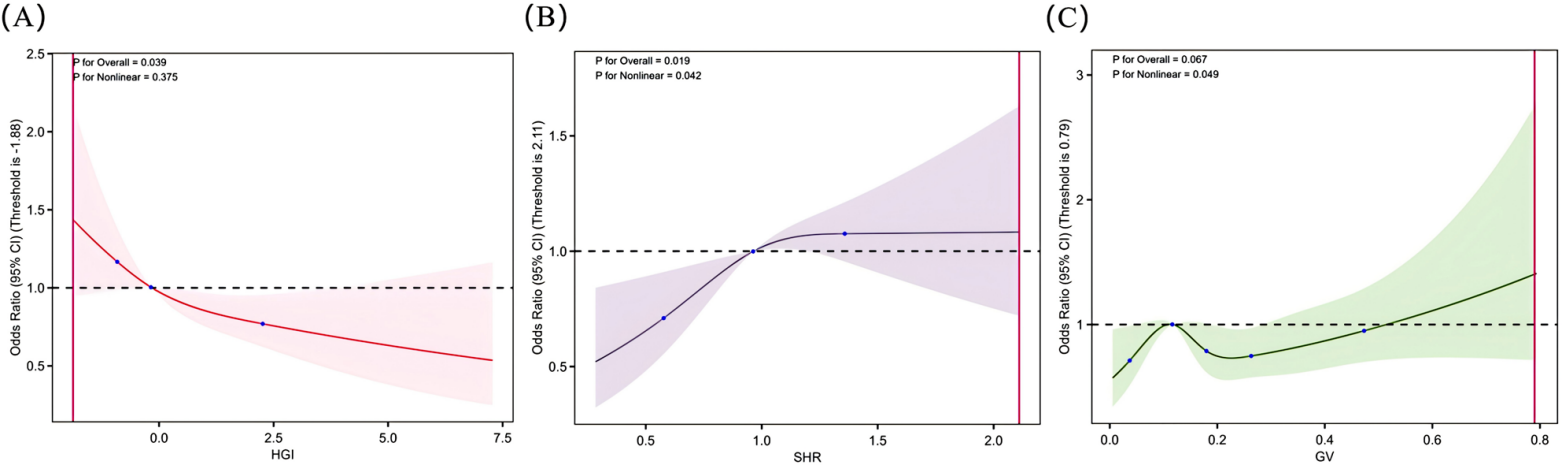
Restricted cubic spline of HGI, SHR, GV and POAF **(A)** HGI (**B**) SHR (**C**) GV

### Nonlinear relationships between glycemic indicators and POAF

To further explore the nonlinear relationship between glucose-related markers and POAF, we conducted a multivariate logistic regression model using RCS analysis (Fig. 2), combined with threshold effect analysis (Supplementary Tables S3-5). As shown in Figure A, HGI showed a significant negative linear relationship with the risk of POAF (P for overall = 0.039; P for nonlinearity = 0.375). The risk of POAF increased significantly at lower HGI values, then gradually decreased with increasing HGI and reached a plateau. A nonlinear relationship was observed between SHR and POAF (P for nonlinearity = 0.042). When SHR was below 0.9067, the risk of POAF increased with increasing SHR. When SHR exceeded 0.9067, the association tended to plateau and the risk no longer increased substantially. GV showed a nonlinear relationship with POAF (P for nonlinearity = 0.049), with no effect at low levels, but once it exceeded 47, the risk of POAF increased sharply.

**Fig. 3:**
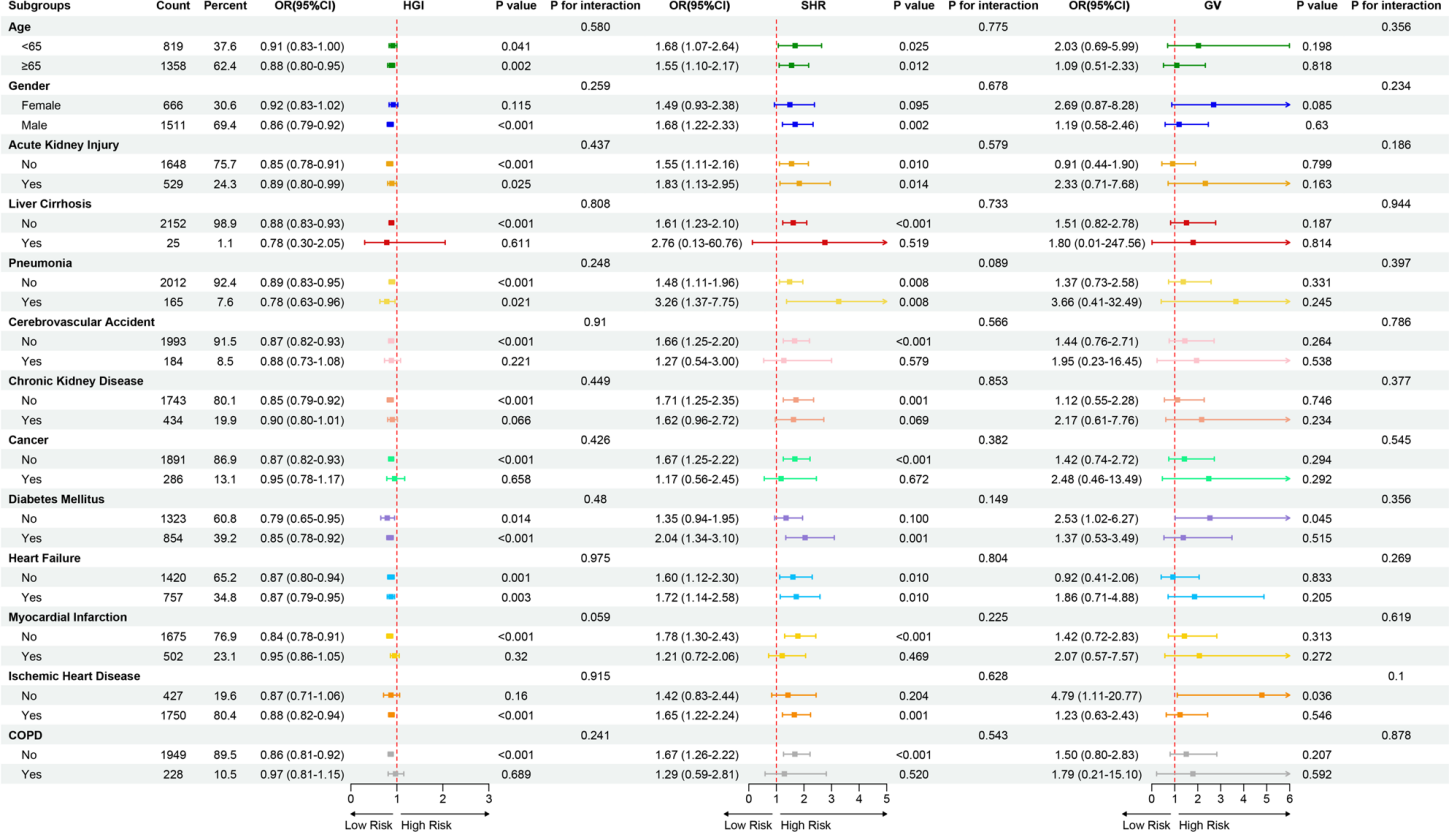
A forest plot depicting the relationship among HGI, SHR, GV, and POAF across various subgroups

### Subgroup analyses

To further verify the stability of SHR, HGI, and GV in predicting POAF across populations with different clinical characteristics (Fig. 3), we performed subgroup analyses and evaluated potential interactions (P for interaction). SHR was significantly positively associated with POAF risk in most subgroups, and common comorbidities such as chronic kidney disease, heart failure, and coronary artery disease did not show significant interaction effects, indicating that SHR remained a relatively stable predictor across diverse clinical contexts. Similarly, HGI demonstrated a consistent protective association, with higher HGI levels associated with lower POAF risk, and no significant interactions were observed, suggesting broad stability of its predictive value.

**Fig. 4:**
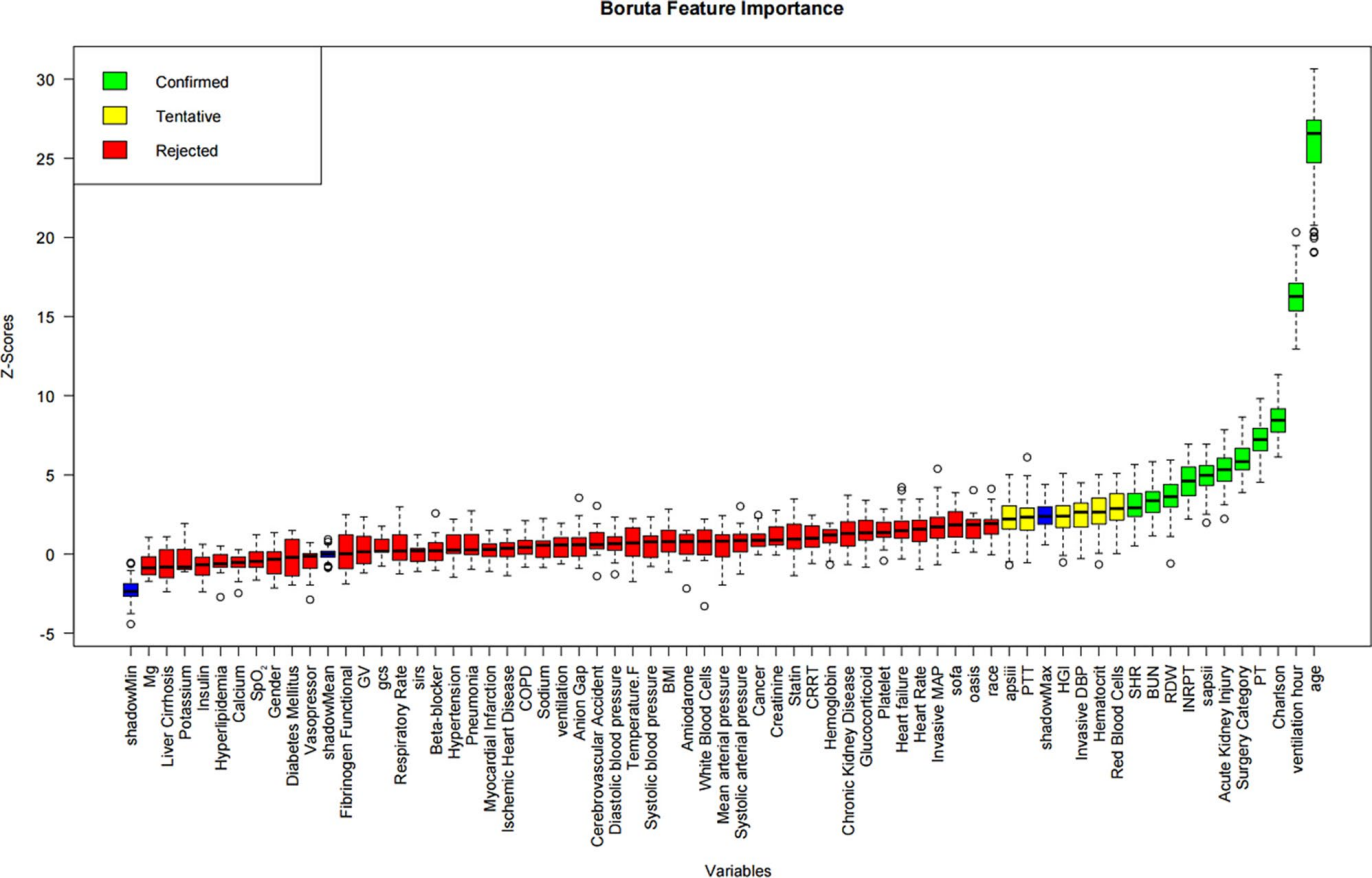
Feature selection for POAF using the boruta algorithm

SHR and HGI showed robust and complementary predictive directions across multiple subgroups, whereas GV exhibited relatively weaker overall predictive performance and appeared more susceptible to individual clinical characteristics, including hypertension status. Further stratified analyses according to diabetes status were conducted to explore predictive consistency across different metabolic backgrounds (Fig. 4). SHR demonstrated a stronger association with POAF among patients with diabetes (OR = 2.04, 95% CI: 1.34–3.10, *P* = 0.001), whereas the association was attenuated and not statistically significant among those without diabetes (OR = 1.35, 95% CI: 0.94–1.95, *P* = 0.100); however, the interaction test was not statistically significant (P for interaction = 0.149). HGI maintained a protective association in both diabetic and non-diabetic subgroups without significant interaction effects (P for interaction = 0.48). GV showed a significant association with POAF only among patients with diabetes (OR = 2.53, 95% CI: 1.02–6.27, *P* = 0.045), although the interaction analysis did not reach statistical significance (P for interaction = 0.356). Overall, these findings suggest that SHR and HGI represent relatively stable predictors across clinical and metabolic contexts, whereas the predictive role of GV may be more context-dependent.

### Sensitivity analyses

First, we conducted a sensitivity test on the calculation method of GV, ( 1 ) using blood glucose values within 72 h after surgery to calculate CV and limit the calculation window. Under the above method, the correlation between GV and POAF still did not reach a statistically significant level (Supplementary Table S6). RCS analysis and threshold effect analysis showed that there was a nonlinear relationship between GV within 72 h after surgery and the risk of POAF. When GV < 0.4645, the change in POAF risk was not significant; however, when GV exceeded 0.4645, the risk of POAF increased significantly (OR = 21.76, 95% CI: 1.93-311.92, *P* = 0.016). The model fit test showed that the threshold model was better than the simple linear model (*P* = 0.015), supporting the existence of a significant threshold effect between GV and POAF ( Supplementary Figure S1, Supplementary Table S7 ) [2]. Excluding GVs with less than three glucose measurements, multivariate logistic regression analysis and RCS analysis were performed. Under the above methods, the correlation between GV and POAF still did not reach a statistically significant level (Supplementary Table S8, Supplementary Figure S2).

The above sensitivity analysis further verified the robustness of the main research conclusions, emphasized the independence and wide applicability of SHR and HGI as predictors of POAF, and also suggested that individual metabolic background and variable selection are of great significance for the establishment of risk assessment models.

### Machine learning model development and performance

The features incorporated into the machine learning model were identified using the Boruta feature selection method (Fig. 4), in which the variables are displayed from right to left in decreasing order of importance. This approach ultimately identified 11 variables as the most relevant predictors of POAF: age, duration of mechanical ventilation, Charlson index, PT, type of surgery, AKI, BUN, RDW, sapsii, SHR, and INR.

To further improve the ability to predict the risk of POAF, we constructed a prediction model based on multiple machine learning methods and optimized and validated it using multi-step feature selection, cross-validation, and interpretability analysis.

**Fig. 5:**
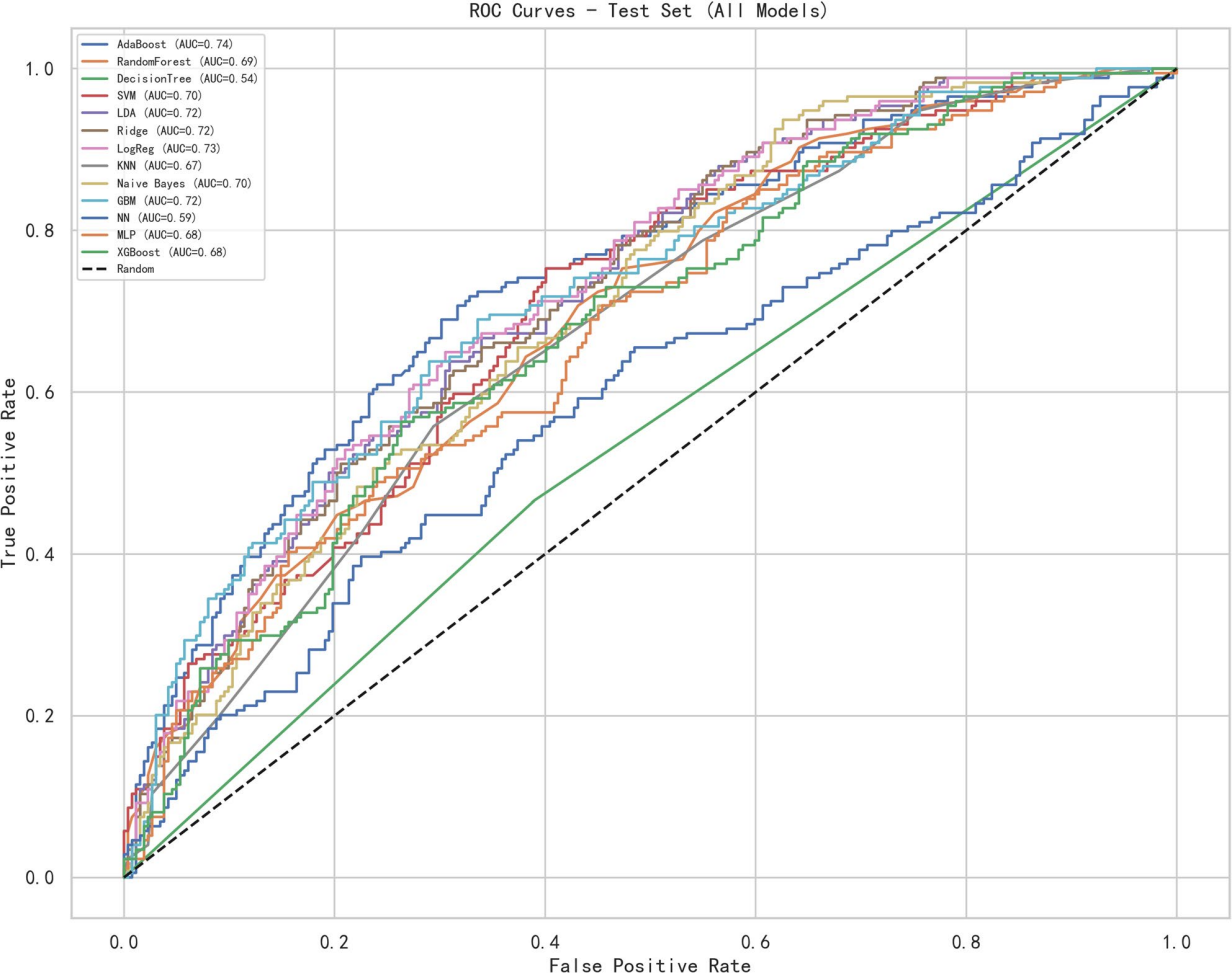
Comparison of ROC curves for machine learning models on the test set

Subsequently, we incorporated these key features and constructed 13 machine learning models, including logistic regression, random forest, support vector machine, gradient boosting machine (GBM), AdaBoost, XGBoost, and neural network (MLP), to predict POAF and evaluate model performance (Fig. 5). The training and validation sets for the models are provided in the Supplementary Materials (Supplementary Table S9). Detailed sensitivity, specificity, and accuracy metrics for all models are provided in the Supplementary Materials (Supplementary Table S10).

After five-fold cross-validation, the AdaBoost model performed best in the test set (AUC = 0.74, sensitivity 85 ). The model demonstrated moderate predictive performance and may provide potential value for risk stratification in future studies.

### Model interpretation (SHAP)

To further explain the model’s predictive mechanism, we created a SHAP explanatory plot (Supplementary Figure S3) based on the AdaBoost model to demonstrate the marginal contribution of each variable to the prediction results. The results showed that age, ventilation hour, surgery category, PT, SHR, AKI, BUN, and RDW were important predictors of the model. The SHAP values for SHR were relatively concentrated, indicating a consistent positive impact on the prediction results. This further validates the important predictive role of SHR as a robust marker of postoperative metabolism and inflammation.

**Fig. 6:**
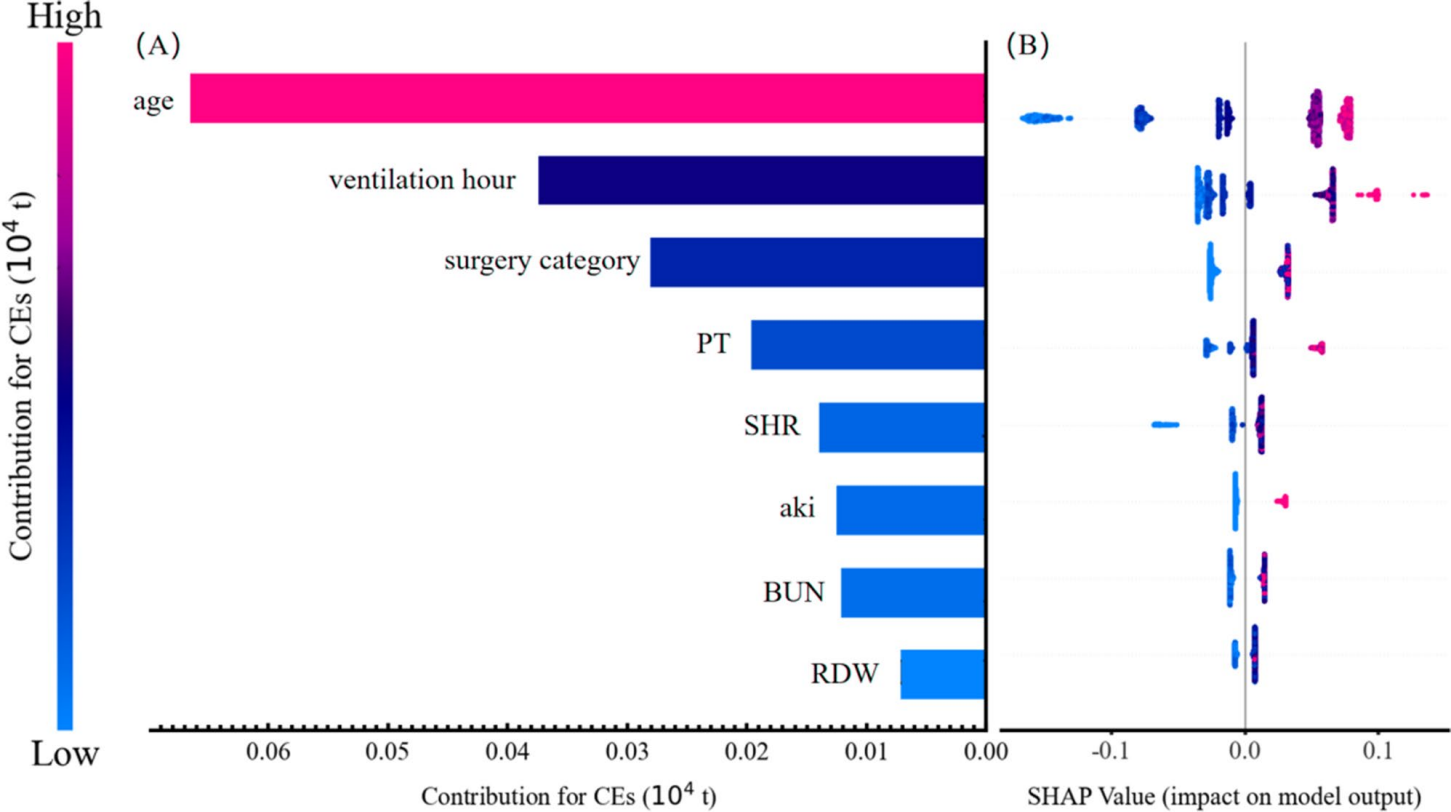
SHAP summary plot of the final Adaboost model

However, three variables in the initial model (sapsii, charlson, and PT-INR) showed minimal predictive contribution in the SHAP plot (average SHAP value approximately 0)(Supplementary Figure S3), so they were removed. After removing these variables, we retrained the machine learning model. The final model variables were: age, ventilation hour, surgery category, PT, SHR, aki, BUN, and RDW. After 5-fold cross-validation, model performance remained unchanged after these removals (AUC = 0.74) (Table 3), and the model structure became more concise. SHR was identified as a significant predictor in the SHAP analysis (Fig. 6).

### Model calibration and clinical utility

**Table 3:** The performance comparison of adaboost model in predicting POAF

	Recall(Sensitivity)	Precision	F1	Specificity	Accuracy	ROC_AUC
Adaboost	0.68	0.68	0.65	0.88	0.68	0.74

Table 3 presents the performance metrics of the AdaBoost model. The model achieved an AUC of 0.74, indicating moderate discriminative ability. Sensitivity was 0.68 and specificity was 0.88, suggesting that the model demonstrates stronger ability to correctly identify patients without POAF while maintaining acceptable detection of high-risk cases.

**Fig. 7:**
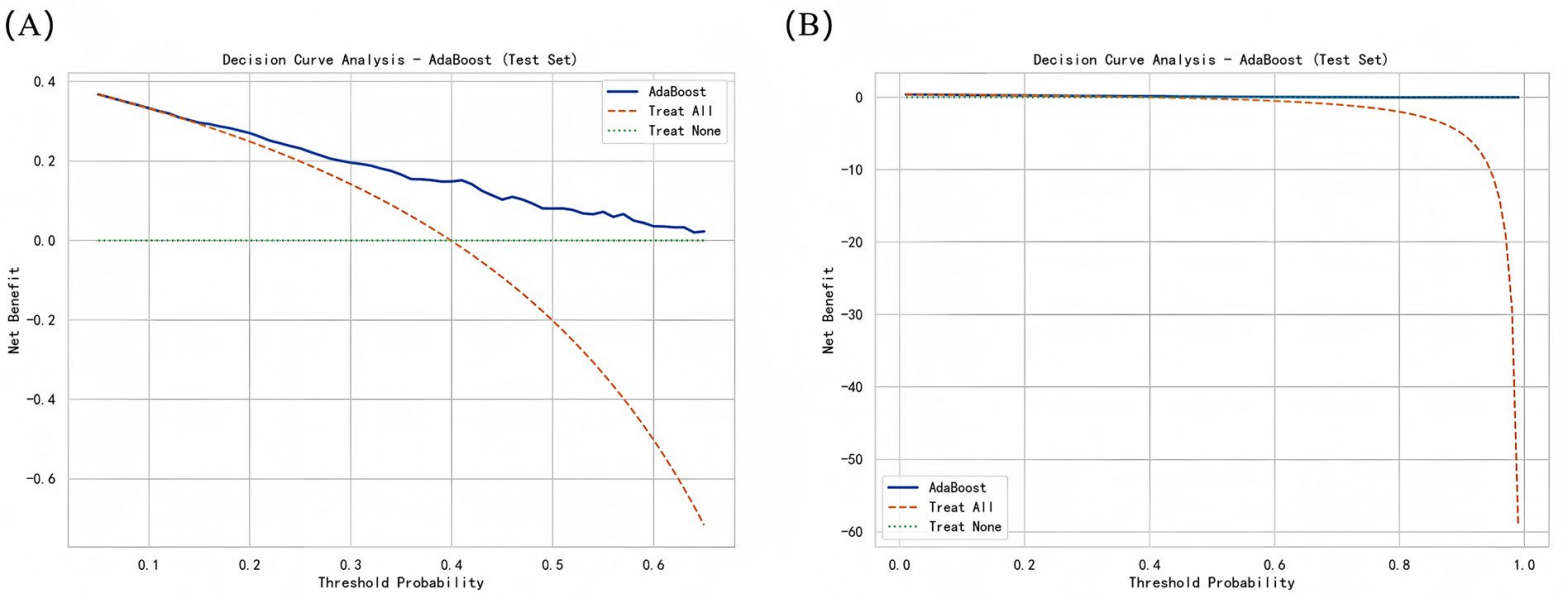
The DCA diagram for the test sets across various threshold probability intervals

Decision curve analysis (Fig. 7) demonstrated that the model provided greater net benefit than treat-all or treat-none strategies across a clinically relevant threshold probability range (approximately 5%–65%). This suggests that within this range, applying the model to guide intensified perioperative monitoring or preventive strategies may yield potential clinical benefit compared with uniform management approaches.

**Fig. 8:**
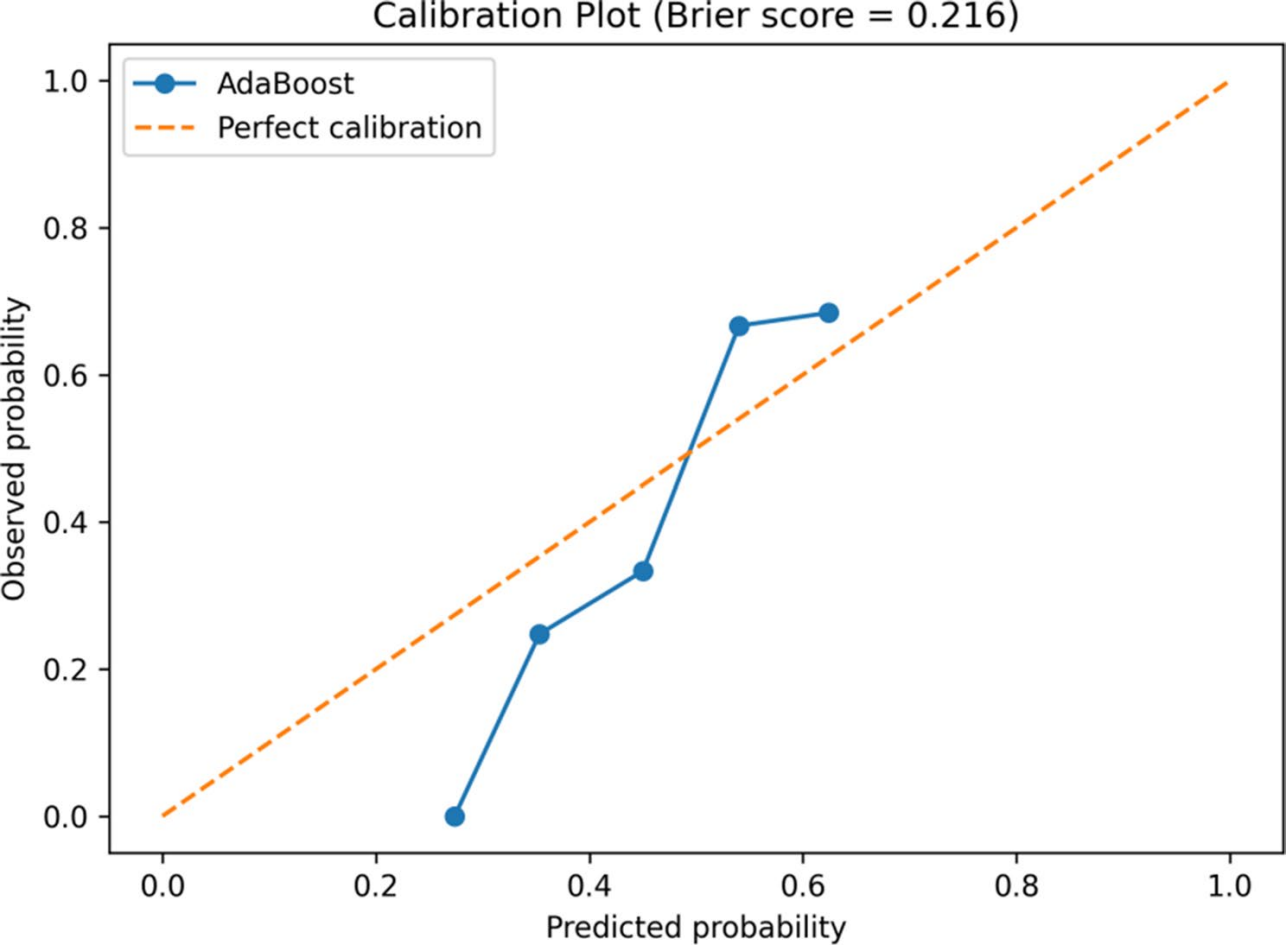
Calibration plot of the AdaBoost model for predicting postoperative atrial fibrillation (POAF)

The calibration plot demonstrated good agreement between predicted probabilities and observed POAF incidence (Fig. 8). Although minor deviations from the ideal diagonal line were observed in certain probability intervals, no substantial systematic miscalibration was identified. The Brier score was 0.216, indicating reasonable overall predictive accuracy.

The calibration plot demonstrated generally acceptable agreement between predicted probabilities and observed POAF incidence (Fig. 8). Although minor deviations from the ideal diagonal line were observed in certain probability ranges, the overall calibration performance was satisfactory. The Brier score was 0.216, indicating reasonable overall predictive accuracy.

The calibration curve shows the agreement between predicted probabilities and observed POAF incidence. The dashed line represents perfect calibration. The Brier score was 0.216.

### Model performance and comparative analysis

The final AdaBoost model achieved an AUC of 0.74 (95% CI 0.684–0.778), indicating moderate discriminative ability.

For comparison, a single-variable logistic regression model constructed using SHR alone yielded an AUC of 0.557 (95% CI 0.512–0.602), which was only slightly above random discrimination.

These findings suggest that integrating multidimensional clinical variables substantially improves predictive performance compared with relying solely on a single metabolic marker.

### Web-based risk prediction tool

Based on our predictive model, we developed the POAF Risk Prediction System as a web tool to enhance its clinical accessibility and ease of use [20]. The access address is: https://heruyi.shinyapps.io/adaBoost/. The screenshots of the website are provided in the supplementary materials (Supplementary Figure S4).

## Discussion

This study systematically evaluated the predictive value of SHR, HGI, and GV for POAF and demonstrated that SHR and HGI are stable independent predictors, while GV showed a nonlinear association with POAF, with risk increasing markedly beyond a threshold of 47. Based on Boruta feature selection and an AdaBoost machine learning algorithm, we developed a prediction model with moderate discriminative performance and implemented it as a web-based prototype to facilitate exploratory evaluation of POAF risk. SHAP analysis further highlighted the importance of SHR as a key predictor, suggesting its potential role as a marker of perioperative stress-related metabolic dysregulation associated with atrial electrical remodeling. Incorporating SHR into perioperative risk assessment may improve the accuracy of POAF risk stratification and support more individualized monitoring and management strategies. Overall, the proposed model provides an exploratory framework for identifying patients at elevated risk of POAF and may support future risk stratification research in perioperative care.

SHR, as an indicator of relative blood glucose elevation under acute stress conditions, has been shown to be associated with a variety of postoperative adverse events [21, 22]. Its increase not only reflects the degree of sympathetic nerve activation, cortisol elevation, and insulin resistance, but is also likely to induce atrial myocardial remodeling through oxidative stress, endothelial dysfunction, and proinflammatory pathways (such as IL-6, TNF-α) [23, 24], increasing the risk of POAF. The robustness of SHR’s predictive value was confirmed in this study through restricted cubic spline (RCS) modeling and SHAP interpretation, aligning with findings from prior mechanistic research. Evidence from earlier investigations has indicated that, among critically ill patients with atrial fibrillation, elevated SHR is strongly linked to increased all-cause mortality at 30, 90, 180, and 365 days. These results suggest that SHR may serve as a valuable marker for evaluating disease severity in ICU patients with atrial fibrillation and for informing therapeutic decision-making [8]. Furthermore, investigations in patients undergoing CABG have shown that elevated SHR is strongly associated with a higher likelihood of POAF [25]. More recent cohort studies have further verified that SHR serves as an independent prognostic factor for early mortality—occurring during hospitalization or within 28 days—in individuals with cardiovascular disease [26].

HGI reveals individual differences in long-term glycation capacity, reflecting the enhanced degree of non-enzymatic glycation under chronic hyperglycemia. Its independent correlation with POAF suggests that long-term abnormal glucose metabolism may potentially promote the electrical structural remodeling of atrial fibrillation through mechanisms such as the AGEs-RAGE pathway, chronic low-grade inflammation, and coronary microperfusion damage [27]. HGI, as the deviation between HbA1c and mean blood glucose, reveals an individual’s susceptibility to glycation. Earlier research has demonstrated a strong link between elevated HGI and the occurrence of adverse cardiovascular outcomes, such as coronary artery disease, stroke, and peripheral arterial disease [28]. Among individuals with severe coronary artery disease, a reduced HGI poses a greater risk to patients with coronary heart disease than an elevated HGI and shows a strong association with both short-term and long-term mortality [29]. Similarly, this study observed that the risk of POAF increased when HGI was at a lower level. This phenomenon is not a paradox. First, large population studies have confirmed that HGI is associated with outcomes in a U-shaped or J-shaped manner: in ≈ 18,000 patients with coronary artery disease(CAD) and diabetes, cardiovascular mortality was significantly increased in the low HGI group [11]. Wen et al. prospectively followed up > 10,000 CAD patients and found that both high and low HGI independently predicted Major Adverse Cardiovascular Events(MACEs) [30]. Low HGI reflects low HbA1c and elevated stress blood glucose on admission, suggesting a strong sympathetic-inflammatory storm, which is prone to induce atrial electrical remodeling through oxidative stress and Ca^2+^ overload. Secondly, red blood cell transfusion during/after cardiac surgery can dilute HbA1c and further lower HGI, and RBC transfusion itself has been shown to be an independent risk factor for POAF [31, 32]. In addition, high inflammation-high RBC turnover states such as anemia, chronic kidney disease, and infection also reduce HbA1c and increase susceptibility to atrial fibrillation. This study incorporated HGI into the POAF risk prediction model for the first time and found it to be an independent protective factor, expanding its application scenarios in perioperative risk assessment.

Prior evidence indicates that, in critically ill patients with atrial fibrillation admitted to the ICU, elevated GV is strongly correlated with increased all-cause mortality across short-, intermediate-, and long-term follow-up periods [9]. In addition, increased GV may also predict the risk of atrial fibrillation in the adult population [33]. Zhou et al., based on a study of patients after cardiac surgery, showed that GV and POAF were incrementally associated. When CV < 24, the risk increased with increasing GV, but it tended to plateau after ≥ 24 [34]. Clement et al.‘s multicenter CABG study also demonstrated that every 10 increase in 24 h-CV increased the risk of POAF by 16 [35]. In contrast, the association between GV and POAF in this study showed a “threshold effect”, and the risk of POAF increased rapidly only when GV > 47. The current research results on the association between GV and POAF are inconsistent, suggesting that GV is less stable in predicting this specific outcome. Possible reasons include: [1] The GV calculation window only covers the postoperative period and does not reflect the overall fluctuations before or during the perioperative period; [2] The mechanism of POAF is mainly intraoperative injury and postoperative inflammation, and the role of GV is relatively indirect; [3] Perioperative insulin intervention may have narrowed the differences between GV groups, resulting in a weakening of its predictive efficacy [4]. GV itself is susceptible to multiple clinical and technical factors (such as blood glucose measurement frequency, selection of observation time window, and metabolic differences between individuals). In summary, the predictive stability of GV for POAF is affected by multiple factors such as indicator selection, monitoring window and SHR collinearity; however, when the fluctuation amplitude reaches ≈ 47 CV, it still suggests the need for strengthened blood glucose-inflammation combined intervention. Future studies should focus on multiple indicators, real-time monitoring and mechanism experiments to clarify the independent and incremental value of GV in POAF risk stratification. In contrast, indicators such as SHR show more consistent and reliable performance in POAF prediction, with higher clinical interpretability and practical value. Future studies incorporating continuous glucose monitoring (CGM) technologies may allow more precise characterization of perioperative glycemic fluctuations, which could help clarify the independent contribution and temporal dynamics of GV in POAF prediction.

With the deepening of understanding of the multidimensional characteristics of blood glucose metabolism, the prognostic value of the combined evaluation of GV, SHR and HGI has gradually attracted attention, especially showing better predictive performance in people with high cardiovascular risk. The combined evaluation of SHR and GV has been proven to be an important tool for predicting Atherosclerotic Cardiovascular Disease(ASCVD) mortality. Compared with a single indicator, it can provide higher predictive accuracy, especially for normal glucose regulation(NGR) and prediabetes(Pre-DM) patients. This comprehensive evaluation method helps to formulate individualized blood glucose management strategies, which is expected to improve clinical outcomes [36]. In critically ill transcatheter aortic valve replacement(TAVR) patients, blood glucose control indicators such as HGI, SHR and GV are closely related to long-term all-cause mortality, providing prognostic information beyond traditional blood glucose indicators. These findings emphasize the potential value of implementing individualized blood glucose management strategies in the TAVR population for improving prognosis [37].

From a model performance perspective, the observed AUC of 0.74 should be interpreted as moderate discrimination. However, previously reported POAF risk scores have demonstrated AUC values ranging approximately from 0.65 to 0.72, suggesting that the present model achieves comparable or slightly improved performance while incorporating dynamic metabolic indicators. The AdaBoost model, integrating the synergistic effects of multiple variables, achieved a higher AUC compared with any single predictor. The mean SHAP value ranking identified age, ventilation hour, surgery category, PT, SHR, AKI, BUN, and RDW as key contributors, with SHR demonstrating stable global importance across predictions. Overall, SHR and HGI demonstrated consistent predictive value across regression analyses, nonlinear modeling, and machine learning interpretation, whereas GV showed a weaker and threshold-dependent association.

The AdaBoost algorithm, by integrating nonlinear relationships and interactions among perioperative variables, demonstrated improved performance compared with individual predictors, while remaining complementary to conventional risk assessment approaches alone. However, the machine learning model should be viewed as a complementary tool rather than a replacement for conventional risk assessment approaches. Traditional clinical judgment and established risk scores remain essential, and the current model primarily provides an exploratory framework for integrating metabolic parameters into risk stratification.

Interpretation of model-derived predictors requires careful distinction between associative importance and causal mechanisms. Variables identified through SHAP analysis, including ventilation duration and surgery category, likely reflect illness severity and perioperative complexity rather than independent pathophysiological drivers of POAF. Similarly, although SHR and HGI showed consistent associations supported by biologically plausible pathways, the observational nature of this study precludes causal inference. Mechanistic interpretations should therefore be regarded as hypothesis-generating rather than definitive conclusions.

The web-based implementation of the AdaBoost model is intended as a research-oriented prototype designed to facilitate exploratory evaluation and hypothesis generation. External validation in independent cohorts and prospective assessment are necessary before considering clinical deployment or integration into decision-support systems.

Overall, the present findings highlight the potential role of machine learning approaches in capturing complex multidimensional relationships among metabolic and perioperative variables. Rather than replacing traditional models, such approaches may complement existing strategies by improving interpretability and identifying novel candidate predictors for future investigation.

Future investigations focusing on dynamic monitoring of SHR during the perioperative period may help identify patients with heightened neuroendocrine stress responses, insulin resistance, and inflammation-driven metabolic dysregulation. These processes may promote atrial electrical remodeling, oxidative stress, and autonomic imbalance, thereby increasing susceptibility to POAF. Consistent with emerging evidence, dynamic metabolic fluctuations may better reflect perioperative physiological instability than single-point measurements.

Beyond glycemic-related parameters, accumulating evidence suggests that multiple clinical and biological factors may contribute to atrial fibrillation (AF) risk prediction and could be incorporated into future artificial intelligence–based models to enhance performance and clinical applicability. Anthropometric and metabolic indicators, particularly measures of central obesity such as waist circumference and body surface area, have demonstrated significant associations with incident AF, highlighting the importance of structural and metabolic substrates in atrial remodeling [38]. Large cohort analyses further suggest that cumulative burden of obesity-related parameters may provide more robust risk stratification compared with single time-point measurements, emphasizing the potential value of longitudinal dynamic monitoring in predictive modeling [39]. Similarly, population-based studies indicate that elevated body mass index and waist circumference levels are independently associated with increased AF risk, supporting the integration of multidimensional metabolic markers into prediction frameworks [40].

In addition to metabolic and anthropometric predictors, emerging data suggest that neurohormonal modulation and cardiac structural remodeling also influence arrhythmia susceptibility. Pharmacological and mechanistic investigations have highlighted the roles of cardiac remodeling pathways and electrophysiological alterations in AF occurrence, suggesting that combining metabolic, inflammatory, structural, and electrophysiological variables may substantially improve risk discrimination in AI-driven prediction tools [41, 42]. Therefore, future machine learning models integrating glycemic indices such as SHR and HGI with broader cardiometabolic and structural biomarkers may enable more precise individualized risk assessment and advance the concept of multidimensional AF prediction.

The present findings have several important clinical implications. First, SHR demonstrated consistent predictive stability and high interpretability, suggesting that dynamic perioperative monitoring could enable early identification of patients at elevated POAF risk. Future studies may explore the potential integration of the web-based AdaBoost tool into electronic medical record systems to assist risk stratification, allowing targeted interventions such as early insulin therapy, anti-inflammatory strategies, or optimization of metabolic status. Second, the model uses readily available perioperative variables and does not require complex or invasive measurements, making it feasible for routine implementation across diverse surgical and critical care settings. Nevertheless, external validation in independent cohorts remains essential before broad clinical implementation.

In recent years, machine learning–based models have increasingly been explored as complementary tools to conventional statistical approaches in medical prediction tasks. Compared with traditional regression-based risk scores, artificial intelligence–driven models such as AdaBoost are capable of capturing nonlinear relationships and complex interactions among multidimensional perioperative variables. In cardiology, machine learning approaches have shown promise in atrial fibrillation prediction, heart failure risk stratification, and assessment of postoperative complications. Unlike conventional scoring systems that rely on fixed coefficients and predefined variables, AI-based models provide greater flexibility and scalability, particularly when integrated into electronic medical record systems. Nevertheless, challenges related to interpretability, transparency, and external validation remain key issues that must be addressed before widespread clinical adoption.

This study has several limitations. Although multivariable adjustment was performed, residual confounding due to baseline imbalances (such as age, SOFA score, and type of surgery) cannot be entirely excluded. Future studies may consider propensity score matching or inverse probability weighting to enhance comparability between groups and strengthen causal inference. Although propensity score matching was considered, we prioritized retaining the full cohort to preserve statistical power and maintain the complete dataset for machine learning model development and evaluation; therefore, multivariable adjustment was applied as the primary approach for confounding control. First, the data were derived from the single-center MIMIC-IV critical care database, and no external validation cohort was included, which may limit the generalizability of the findings. Second, key intraoperative variables, including cardiopulmonary bypass time and aortic cross-clamp duration, were unavailable and therefore not incorporated into the analysis, potentially omitting relevant confounders. Third, as a retrospective observational study, causal relationships between glycemic parameters and POAF cannot be established. Fourth, heterogeneity in postoperative blood glucose measurement frequency may have introduced information bias. Finally, POAF episodes were not stratified by duration (e.g., transient versus sustained), which may have masked potential differences in risk profiles and prognostic implications.

Future research should prioritize external validation in multicenter cohorts (e.g., eICU or independently established datasets) and incorporate key intraoperative parameters, such as cardiopulmonary bypass time and aortic cross-clamp duration, to further optimize predictive performance. Prospective interventional studies are needed to determine whether risk stratification based on SHR, HGI, and GV can guide individualized perioperative glucose management and potentially reduce the incidence of POAF. Moreover, integrating these glycemic indices with inflammatory biomarkers, oxidative stress profiles, and advanced cardiac imaging modalities may help elucidate the mechanistic pathways linking metabolic dysregulation to atrial electrical remodeling. Such efforts may further clarify the mechanistic links between metabolic dysregulation and atrial electrical remodeling.

## Conclusion

In this large critical care cohort, SHR and HGI were stable, independent predictors of POAF, with HGI showing an inverse linear association and SHR displaying a threshold effect: risk rose sharply when SHR < 0.9067 but plateaued thereafter. GV showed a similar threshold, with POAF risk increasing above 47. The AdaBoost model achieved moderate discrimination (AUC = 0.74). The model was implemented as a web-based prototype to facilitate exploratory evaluation of POAF risk. Together, SHR, HGI, and GV reflect complementary aspects of perioperative glucose metabolism, offering novel opportunities for POAF risk stratification and individualized management. External validation in independent cohorts is required before clinical implementation.

## Supplementary Information

Below is the link to the electronic supplementary material.

Supplementary Material 1

## Data Availability

The dataset underpinning the results of this research is derived from MIMIC-IV and is publicly available through the PhysioNet platform at https://physionet.org/content/mimiciv/3.1/.

## Abbreviations

POAF: Postoperative atrial fibrillation
SHR: Stress hyperglycemia ratio
GV: Glycemic variability
HGI: Hemoglobin glycation index
RCS: Restricted cubic splines
AUC: Area under the curve
SHAP: SHapley Additive exPlanations
CITI: Collaborative Institutional Training Initiative
MIMIC-IV: Medical Information Mart for Intensive Care IV
STROBE: Strengthening the Reporting of Observational Studies in Epidemiology
SQL: Structured query language
COPD: Chronic obstructive pulmonary disease
CCI: Charlson Comorbidity Index
SOFA: Sequential Organ Failure Assessment
APSIII: Acute Physiology Score III
SAPS-II: Simplified Acute Physiology Score II
OASIS: Oxford Acute Severity of Disease Score
SIRS: Systemic Inflammatory Response Syndrome
GCS: Glasgow Coma Scale
SBP: Systolic blood pressure
DBP: Diastolic blood pressure
MBP: Mean blood pressure
ABPs: Arterial blood pressure systolic
ABPd: Arterial blood pressure diastolic
ABPm: Mean arterial blood pressure
SpO₂: Mean pulse oxygen saturation
AKI: Acute kidney injury
Temperature F: Body temperature (Fahrenheit)
RBC: Red blood cells
WBC: White blood cells
PLT: Platelet count
RDW: Red cell distribution width
ANC: Neutrophil count
ALC: Lymphocyte count
ALB: Albumin
GLB: Globulin
TP: Total protein
BUN: Urea nitrogen / Blood urea nitrogen
UA: Uric acid
ALT: Alanine aminotransferase
AST: Aspartate aminotransferase
LDH: Lactate dehydrogenase
TBIL: Total bilirubin
DBIL: Direct bilirubin
IBIL: Indirect bilirubin
CK: Creatine kinase
CK-MB: Creatine kinase MB isoenzyme
NTproBNP: N-terminal pro-B-type natriuretic peptide
TnI: Troponin I
TnT: Troponin T
FIB: Fibrinogen functional
DD: D-dimer
PT: Prothrombin time
PTT: Partial thromboplastin time
INR: International normalized ratio
THR: Thrombin
TC: Total cholesterol
TG: Triglycerides
HDL-C: High-density lipoprotein cholesterol
LDL-C: Low-density lipoprotein cholesterol
CRRT: Continuous renal replacement therapy
ECG: Electrocardiogram
VIF: Variance inflation factor
CABG: Coronary artery bypass grafting
ICU: Intensive care unit
GBM: Gradient boosting machine
MLP: Multilayer perceptron neural network
DCA: Decision curve analysis
CAD: Coronary artery disease
TAVR: Transcatheter aortic valve replacement
ASCVD: Atherosclerotic Cardiovascular Disease
NGR: Normal glucose regulation
Pre-DM: Prediabetes
