# Supplementary material for: Prognostic value of stress hyperglycemia ratio, hemoglobin glycation index, and glycemic variability for postoperative atrial fibrillation: a machine learning-based prediction model
Source: BMC Med Inform Decis Mak. 2026 Apr 24;26:208. doi: 10.1186/s12911-026-03507-z (PMC13244604; doi:10.1186/s12911-026-03507-z)

Supplementary Material

Supplementary Table S1. List of ICD Diagnosis Codes Included in the Study

| CABG | ICD9 | 3611, 3612, 3613, 3614, 3615, 3616  3611, 3612, 3613, 3614, 3615, 3616  3611, 3612, 3613, 3614, 3615, 3616 |
| --- | --- | --- |
|  | ICD10 | 021008W, 021009W, 02100AW, 02100W, 02100KW, 021048W, 021049W, 02104A9, 02104AC, 02104AF, 02104AW, 02104D4, 02104J3, 02104J8, 02104J9, 02104JC, 02104JF, 02104JW, 02104K3, 02104K8, 02104K9, 02104KC, 02104KF, 02104KW, 02104Z3, 02104Z8, 02104Z9, 02104ZC, 02104ZF, 0211083, 0211088, 0211089, 021108C, 021108F, 021108W, 0211093, 0211098, 0211099, 021109C, 021109F, 021109W, 02110A3, 02110A8, 02110A9, 02110AC, 02110AF, 02110AW, 02110J3, 02110J8, 02110J9, 02110JC, 02110JF, 02110JW, 02110K3, 02110K8, 02110K9, 02110KC, 02110KW, 02110Z3, 02110Z8, 02110Z9, 02110ZC, 02110ZF, 0211344, 02113D4, 0211444, 0211483, 0211488, 02120K8, 02120K9, 02120KC, 02120KF, 02120KW, 02120Z3, 02120Z8, 02120Z9, 02120ZC, 02120ZF, 0212344, 02123D4, 0212444, 0212483, 0212488, 0212489, 021248C, 021248F, 021248W, 0212493, 0212498, 0212499, 021249C, 021249F, 021249W, 02124A3, 02124A8, 02124A9, 02124AC, 02124AF, 02124AW, 02124D4, 02124J3, 02124J8, 02124J9, 02124JC, 02124JF, 02124JW, 02124K3, 02124K8, 02124K9, 02124KC, 02124KF, 02124KW, 02124Z3, 02124Z8, 02124Z9, 02124ZC, 02124ZF, 0213083, 0213088, 0213089, 021308C, 021308F, 021308W, 0213093, 0213098, 0213099, 021309W, 02130AW, 021008W, 021009W, 02100AW, 02100W, 02100KW, 021048W, 021049W, 02104A9, 02104AC, 02104AF, 02104AW, 02104D4, 02104J3, 02104J8, 02104J9, 02104JC, 02104JF, 02104JW, 02104K3, 02104K8, 02104K9, 02104KC, 02104KF, 02104KW, 02104Z3, 02104Z8, 02104Z9, 02104ZC, 02104ZF, 0211083, 0211088, 0211089, 021108C, 021108F, 021108W, 0211093, 0211098, 0211099, 021109C, 021109F, 021109W, 02110A3, 02110A8, 02110A9, 02110AC, 02110AF, 02110AW, 02110J3, 02110J8, 02110J9, 02110JC, 02110JF, 02110JW, 02110K3, 02110K8, 02110K9, 02110KC, 02110KW, 02110Z3, 02110Z8, 02110Z9, 02110ZC, 02110ZF, 0211344, 02113D4, 0211444, 0211483, 0211488, 02120K8, 02120K9, 02120KC, 02120KF, 02120KW, 02120Z3, 02120Z8, 02120Z9, 02120ZC, 02120ZF, 0212344, 02123D4, 0212444, 0212483, 0212488, 0212489, 021248C, 021248F, 021248W, 0212493, 0212498, 0212499, 021249C, 021249F, 021249W, 02124A3, 02124A8, 02124A9, 02124AC, 02124AF, 02124AW, 02124D4, 02124J3, 02124J8, 02124J9, 02124JC, 02124JF, 02124JW, 02124K3, 02124K8, 02124K9, 02124KC, 02124KF, 02124KW, 02124Z3, 02124Z8, 02124Z9, 02124ZC, 02124ZF, 0213083, 0213088, 0213089, 021308C, 021308F, 021308W, 0213093, 0213098, 0213099, 021309W, 02130AW, 021008W, 021009W, 02100AW, 02100W, 02100KW, 021048W, 021049W, 02104A9, 02104AC, 02104AF, 02104AW, 02104D4, 02104J3, 02104J8, 02104J9, 02104JC, 02104JF, 02104JW, 02104K3, 02104K8, 02104K9, 02104KC, 02104KF, 02104KW, 02104Z3, 02104Z8, 02104Z9, 02104ZC, 02104ZF, 0211083, 0211088, 0211089, 021108C, 021108F, 021108W, 0211093, 0211098, 0211099, 021109C, 021109F, 021109W, 02110A3, 02110A8, 02110A9, 02110AC, 02110AF, 02110AW, 02110J3, 02110J8, 02110J9, 02110JC, 02110JF, 02110JW, 02110K3, 02110K8, 02110K9, 02110KC, 02110KW, 02110Z3, 02110Z8, 02110Z9, 02110ZC, 02110ZF, 0211344, 02113D4, 0211444, 0211483, 0211488, 02120K8, 02120K9, 02120KC, 02120KF, 02120KW, 02120Z3, 02120Z8, 02120Z9, 02120ZC, 02120ZF, 0212344, 02123D4, 0212444, 0212483, 0212488, 0212489, 021248C, 021248F, 021248W, 0212493, 0212498, 0212499, 021249C, 021249F, 021249W, 02124A3, 02124A8, 02124A9, 02124AC, 02124AF, 02124AW, 02124D4, 02124J3, 02124J8, 02124J9, 02124JC, 02124JF, 02124JW, 02124K3, 02124K8, 02124K9, 02124KC, 02124KF, 02124KW, 02124Z3, 02124Z8, 02124Z9, 02124ZC, 02124ZF, 0213083, 0213088, 0213089, 021308C, 021308F, 021308W, 0213093, 0213098, 0213099, 021309W, 02130AW, 021008W, 021009W, 02100AW, 02100W, 02100KW, 021048W, 021049W, 02104A9, 02104AC, 02104AF, 02104AW, 02104D4, 02104J3, 02104J8, 02104J9, 02104JC, 02104JF, 02104JW, 02104K3, 02104K8, 02104K9, 02104KC, 02104KF, 02104KW, 02104Z3, 02104Z8, 02104Z9, 02104ZC, 02104ZF, 0211083, 0211088, 0211089, 021108C, 021108F, 021108W, 0211093, 0211098, 0211099, 021109C, 021109F, 021109W, 02110A3, 02110A8, 02110A9, 02110AC, 02110AF, 02110AW, 02110J3, 02110J8, 02110J9, 02110JC, 02110JF, 02110JW, 02110K3, 02110K8, 02110K9, 02110KC, 02110KW, 02110Z3, 02110Z8, 02110Z9, 02110ZC, 02110ZF, 0211344, 02113D4, 0211444, 0211483, 0211488, 02120K8, 02120K9, 02120KC, 02120KF, 02120KW, 02120Z3, 02120Z8, 02120Z9, 02120ZC, 02120ZF, 0212344, 02123D4, 0212444, 0212483, 0212488, 0212489, 021248C, 021248F, 021248W, 0212493, 0212498, 0212499, 021249C, 021249F, 021249W, 02124A3, 02124A8, 02124A9, 02124AC, 02124AF, 02124AW, 02124D4, 02124J3, 02124J8, 02124J9, 02124JC, 02124JF, 02124JW, 02124K3, 02124K8, 02124K9, 02124KC, 02124KF, 02124KW, 02124Z3, 02124Z8, 02124Z9, 02124ZC, 02124ZF, 0213083, 0213088, 0213089, 021308C, 021308F, 021308W, 0213093, 0213098, 0213099, 021309W, 02130AW, 021008W, 021009W, 02100AW, 02100W, 02100KW, 021048W, 021049W, 02104A9, 02104AC, 02104AF, 02104AW, 02104D4, 02104J3, 02104J8, 02104J9, 02104JC, 02104JF, 02104JW, 02104K3, 02104K8, 02104K9, 02104KC, 02104KF, 02104KW, 02104Z3, 02104Z8, 02104Z9, 02104ZC, 02104ZF, 0211083, 0211088, 0211089, 021108C, 021108F, 021108W, 0211093, 0211098, 0211099, 021109C, 021109F, 021109W, 02110A3, 02110A8, 02110A9, 02110AC, 02110AF, 02110AW, 02110J3, 02110J8, 02110J9, 02110JC, 02110JF, 02110JW, 02110K3, 02110K8, 02110K9, 02110KC, 02110KW, 02110Z3, 02110Z8, 02110Z9, 02110ZC, 02110ZF, 0211344, 02113D4, 0211444, 0211483, 0211488, 02120K8, 02120K9, 02120KC, 02120KF, 02120KW, 02120Z3, 02120Z8, 02120Z9, 02120ZC, 02120ZF, 0212344, 02123D4, 0212444, 0212483, 0212488, 0212489, 021248C, 021248F, 021248W, 0212493, 0212498, 0212499, 021249C, 021249F, 021249W, 02124A3, 02124A8, 02124A9, 02124AC, 02124AF, 02124AW, 02124D4, 02124J3, 02124J8, 02124J9, 02124JC, 02124JF, 02124JW, 02124K3, 02124K8, 02124K9, 02124KC, 02124KF, 02124KW, 02124Z3, 02124Z8, 02124Z9, 02124ZC, 02124ZF, 0213083, 0213088, 0213089, 021308C, 021308F, 021308W, 0213093, 0213098, 0213099, 021309W, 02130AW  021008W, 021009W, 02100AW, 02100W, 02100KW, 021048W, 021049W, 02104A9, 02104AC, 02104AF, 02104AW, 02104D4, 02104J3, 02104J8, 02104J9, 02104JC, 02104JF, 02104JW, 02104K3, 02104K8, 02104K9, 02104KC, 02104KF, 02104KW, 02104Z3, 02104Z8, 02104Z9, 02104ZC, 02104ZF, 0211083, 0211088, 0211089, 021108C, 021108F, 021108W, 0211093, 0211098, 0211099, 021109C, 021109F, 021109W, 02110A3, 02110A8, 02110A9, 02110AC, 02110AF, 02110AW, 02110J3, 02110J8, 02110J9, 02110JC, 02110JF, 02110JW, 02110K3, 02110K8, 02110K9, 02110KC, 02110KW, 02110Z3, 02110Z8, 02110Z9, 02110ZC, 02110ZF, 0211344, 02113D4, 0211444, 0211483, 0211488, 02120K8, 02120K9, 02120KC, 02120KF, 02120KW, 02120Z3, 02120Z8, 02120Z9, 02120ZC, 02120ZF, 0212344, 02123D4, 0212444, 0212483, 0212488, 0212489, 021248C, 021248F, 021248W, 0212493, 0212498, 0212499, 021249C, 021249F, 021249W, 02124A3, 02124A8, 02124A9, 02124AC, 02124AF, 02124AW, 02124D4, 02124J3, 02124J8, 02124J9, 02124JC, 02124JF, 02124JW, 02124K3, 02124K8, 02124K9, 02124KC, 02124KF, 02124KW, 02124Z3, 02124Z8, 02124Z9, 02124ZC, 02124ZF, 0213083, 0213088, 0213089, 021308C, 021308F, 021308W, 0213093, 0213098, 0213099, 021309W, 02130AW, 021008W, 021009W, 02100AW, 02100W, 02100KW, 021048W, 021049W, 02104A9, 02104AC, 02104AF, 02104AW, 02104D4, 02104J3, 02104J8, 02104J9, 02104JC, 02104JF, 02104JW, 02104K3, 02104K8, 02104K9, 02104KC, 02104KF, 02104KW, 02104Z3, 02104Z8, 02104Z9, 02104ZC, 02104ZF, 0211083, 0211088, 0211089, 021108C, 021108F, 021108W, 0211093, 0211098, 0211099, 021109C, 021109F, 021109W, 02110A3, 02110A8, 02110A9, 02110AC, 02110AF, 02110AW, 02110J3, 02110J8, 02110J9, 02110JC, 02110JF, 02110JW, 02110K3, 02110K8, 02110K9, 02110KC, 02110KW, 02110Z3, 02110Z8, 02110Z9, 02110ZC, 02110ZF, 0211344, 02113D4, 0211444, 0211483, 0211488, 02120K8, 02120K9, 02120KC, 02120KF, 02120KW, 02120Z3, 02120Z8, 02120Z9, 02120ZC, 02120ZF, 0212344, 02123D4, 0212444, 0212483, 0212488, 0212489, 021248C, 021248F, 021248W, 0212493, 0212498, 0212499, 021249C, 021249F, 021249W, 02124A3, 02124A8, 02124A9, 02124AC, 02124AF, 02124AW, 02124D4, 02124J3, 02124J8, 02124J9, 02124JC, 02124JF, 02124JW, 02124K3, 02124K8, 02124K9, 02124KC, 02124KF, 02124KW, 02124Z3, 02124Z8, 02124Z9, 02124ZC, 02124ZF, 0213083, 0213088, 0213089, 021308C, 021308F, 021308W, 0213093, 0213098, 0213099, 021309W, 02130AW, 021008W, 021009W, 02100AW, 02100W, 02100KW, 021048W, 021049W, 02104A9, 02104AC, 02104AF, 02104AW, 02104D4, 02104J3, 02104J8, 02104J9, 02104JC, 02104JF, 02104JW, 02104K3, 02104K8, 02104K9, 02104KC, 02104KF, 02104KW, 02104Z3, 02104Z8, 02104Z9, 02104ZC, 02104ZF, 0211083, 0211088, 0211089, 021108C, 021108F, 021108W, 0211093, 0211098, 0211099, 021109C, 021109F, 021109W, 02110A3, 02110A8, 02110A9, 02110AC, 02110AF, 02110AW, 02110J3, 02110J8, 02110J9, 02110JC, 02110JF, 02110JW, 02110K3, 02110K8, 02110K9, 02110KC, 02110KW, 02110Z3, 02110Z8, 02110Z9, 02110ZC, 02110ZF, 0211344, 02113D4, 0211444, 0211483, 0211488, 02120K8, 02120K9, 02120KC, 02120KF, 02120KW, 02120Z3, 02120Z8, 02120Z9, 02120ZC, 02120ZF, 0212344, 02123D4, 0212444, 0212483, 0212488, 0212489, 021248C, 021248F, 021248W, 0212493, 0212498, 0212499, 021249C, 021249F, 021249W, 02124A3, 02124A8, 02124A9, 02124AC, 02124AF, 02124AW, 02124D4, 02124J3, 02124J8, 02124J9, 02124JC, 02124JF, 02124JW, 02124K3, 02124K8, 02124K9, 02124KC, 02124KF, 02124KW, 02124Z3, 02124Z8, 02124Z9, 02124ZC, 02124ZF, 0213083, 0213088, 0213089, 021308C, 021308F, 021308W, 0213093, 0213098, 0213099, 021309W, 02130AW, 021008W, 021009W, 02100AW, 02100W, 02100KW, 021048W, 021049W, 02104A9, 02104AC, 02104AF, 02104AW, 02104D4, 02104J3, 02104J8, 02104J9, 02104JC, 02104JF, 02104JW, 02104K3, 02104K8, 02104K9, 02104KC, 02104KF, 02104KW, 02104Z3, 02104Z8, 02104Z9, 02104ZC, 02104ZF, 0211083, 0211088, 0211089, 021108C, 021108F, 021108W, 0211093, 0211098, 0211099, 021109C, 021109F, 021109W, 02110A3, 02110A8, 02110A9, 02110AC, 02110AF, 02110AW, 02110J3, 02110J8, 02110J9, 02110JC, 02110JF, 02110JW, 02110K3, 02110K8, 02110K9, 02110KC, 02110KW, 02110Z3, 02110Z8, 02110Z9, 02110ZC, 02110ZF, 0211344, 02113D4, 0211444, 0211483, 0211488, 02120K8, 02120K9, 02120KC, 02120KF, 02120KW, 02120Z3, 02120Z8, 02120Z9, 02120ZC, 02120ZF, 0212344, 02123D4, 0212444, 0212483, 0212488, 0212489, 021248C, 021248F, 021248W, 0212493, 0212498, 0212499, 021249C, 021249F, 021249W, 02124A3, 02124A8, 02124A9, 02124AC, 02124AF, 02124AW, 02124D4, 02124J3, 02124J8, 02124J9, 02124JC, 02124JF, 02124JW, 02124K3, 02124K8, 02124K9, 02124KC, 02124KF, 02124KW, 02124Z3, 02124Z8, 02124Z9, 02124ZC, 02124ZF, 0213083, 0213088, 0213089, 021308C, 021308F, 021308W, 0213093, 0213098, 0213099, 021309W, 02130AW, 021008W, 021009W, 02100AW, 02100W, 02100KW, 021048W, 021049W, 02104A9, 02104AC, 02104AF, 02104AW, 02104D4, 02104J3, 02104J8, 02104J9, 02104JC, 02104JF, 02104JW, 02104K3, 02104K8, 02104K9, 02104KC, 02104KF, 02104KW, 02104Z3, 02104Z8, 02104Z9, 02104ZC, 02104ZF, 0211083, 0211088, 0211089, 021108C, 021108F, 021108W, 0211093, 0211098, 0211099, 021109C, 021109F, 021109W, 02110A3, 02110A8, 02110A9, 02110AC, 02110AF, 02110AW, 02110J3, 02110J8, 02110J9, 02110JC, 02110JF, 02110JW, 02110K3, 02110K8, 02110K9, 02110KC, 02110KW, 02110Z3, 02110Z8, 02110Z9, 02110ZC, 02110ZF, 0211344, 02113D4, 0211444, 0211483, 0211488, 02120K8, 02120K9, 02120KC, 02120KF, 02120KW, 02120Z3, 02120Z8, 02120Z9, 02120ZC, 02120ZF, 0212344, 02123D4, 0212444, 0212483, 0212488, 0212489, 021248C, 021248F, 021248W, 0212493, 0212498, 0212499, 021249C, 021249F, 021249W, 02124A3, 02124A8, 02124A9, 02124AC, 02124AF, 02124AW, 02124D4, 02124J3, 02124J8, 02124J9, 02124JC, 02124JF, 02124JW, 02124K3, 02124K8, 02124K9, 02124KC, 02124KF, 02124KW, 02124Z3, 02124Z8, 02124Z9, 02124ZC, 02124ZF, 0213083, 0213088, 0213089, 021308C, 021308F, 021308W, 0213093, 0213098, 0213099, 021309W, 02130AW  021008W, 021009W, 02100AW, 02100W, 02100KW, 021048W, 021049W, 02104A9, 02104AC, 02104AF, 02104AW, 02104D4, 02104J3, 02104J8, 02104J9, 02104JC, 02104JF, 02104JW, 02104K3, 02104K8, 02104K9, 02104KC, 02104KF, 02104KW, 02104Z3, 02104Z8, 02104Z9, 02104ZC, 02104ZF, 0211083, 0211088, 0211089, 021108C, 021108F, 021108W, 0211093, 0211098, 0211099, 021109C, 021109F, 021109W, 02110A3, 02110A8, 02110A9, 02110AC, 02110AF, 02110AW, 02110J3, 02110J8, 02110J9, 02110JC, 02110JF, 02110JW, 02110K3, 02110K8, 02110K9, 02110KC, 02110KW, 02110Z3, 02110Z8, 02110Z9, 02110ZC, 02110ZF, 0211344, 02113D4, 0211444, 0211483, 0211488, 02120K8, 02120K9, 02120KC, 02120KF, 02120KW, 02120Z3, 02120Z8, 02120Z9, 02120ZC, 02120ZF, 0212344, 02123D4, 0212444, 0212483, 0212488, 0212489, 021248C, 021248F, 021248W, 0212493, 0212498, 0212499, 021249C, 021249F, 021249W, 02124A3, 02124A8, 02124A9, 02124AC, 02124AF, 02124AW, 02124D4, 02124J3, 02124J8, 02124J9, 02124JC, 02124JF, 02124JW, 02124K3, 02124K8, 02124K9, 02124KC, 02124KF, 02124KW, 02124Z3, 02124Z8, 02124Z9, 02124ZC, 02124ZF, 0213083, 0213088, 0213089, 021308C, 021308F, 021308W, 0213093, 0213098, 0213099, 021309W, 02130AW |
| Valve | ICD10 | 027F3ZZ, 027G3ZZ, 02BG3ZZ, 02QJ0ZZ, 02RF08Z, 02RF37Z, 02RF38H, 02RF38Z, 02RF3JZ, 02RF3KZ, 02RG08Z, 02UU3JZ, 02WF38Z, 02WF3JZ, 02WG38Z, 02WG3JZ, X2RF032, X2RF332, X2RF432, 027F3ZZ, 027G3ZZ, 02BG3ZZ, 02QJ0ZZ, 02RF08Z, 02RF37Z, 02RF38H, 02RF38Z, 02RF3JZ, 02RF3KZ, 02RG08Z, 02UU3JZ, 02WF38Z, 02WF3JZ, 02WG38Z, 02WG3JZ, X2RF032, X2RF332, X2RF432, 027F3ZZ, 027G3ZZ, 02BG3ZZ, 02QJ0ZZ, 02RF08Z, 02RF37Z, 02RF38H, 02RF38Z, 02RF3JZ, 02RF3KZ, 02RG08Z, 02UU3JZ, 02WF38Z, 02WF3JZ, 02WG38Z, 02WG3JZ, X2RF032, X2RF332, X2RF432, 027F3ZZ, 027G3ZZ, 02BG3ZZ, 02QJ0ZZ, 02RF08Z, 02RF37Z, 02RF38H, 02RF38Z, 02RF3JZ, 02RF3KZ, 02RG08Z, 02UU3JZ, 02WF38Z, 02WF3JZ, 02WG38Z, 02WG3JZ, X2RF032, X2RF332, X2RF432, 027F3ZZ, 027G3ZZ, 02BG3ZZ, 02QJ0ZZ, 02RF08Z, 02RF37Z, 02RF38H, 02RF38Z, 02RF3JZ, 02RF3KZ, 02RG08Z, 02UU3JZ, 02WF38Z, 02WF3JZ, 02WG38Z, 02WG3JZ, X2RF032, X2RF332, X2RF432, 027F3ZZ, 027G3ZZ, 02BG3ZZ, 02QJ0ZZ, 02RF08Z, 02RF37Z, 02RF38H, 02RF38Z, 02RF3JZ, 02RF3KZ, 02RG08Z, 02UU3JZ, 02WF38Z, 02WF3JZ, 02WG38Z, 02WG3JZ, X2RF032, X2RF332, X2RF432, 027F3ZZ, 027G3ZZ, 02BG3ZZ, 02QJ0ZZ, 02RF08Z, 02RF37Z, 02RF38H, 02RF38Z, 02RF3JZ, 02RF3KZ, 02RG08Z, 02UU3JZ, 02WF38Z, 02WF3JZ, 02WG38Z, 02WG3JZ, X2RF032, X2RF332, X2RF432, 027F3ZZ, 027G3ZZ, 02BG3ZZ, 02QJ0ZZ, 02RF08Z, 02RF37Z, 02RF38H, 02RF38Z, 02RF3JZ, 02RF3KZ, 02RG08Z, 02UU3JZ, 02WF38Z, 02WF3JZ, 02WG38Z, 02WG3JZ, X2RF032, X2RF332, X2RF432 |
|  | ICD9 | 3505, 3506, 3511, 3512, 3514, 3521, 3522, 3523, icd9_3524, 3525, 3527, 3528, 3539, 3596, 3597, 3599 |
| Aorta | ICD10 | 02UW08Z, 02UW3JZ, 02UX07Z, 02UX08Z |
|  | ICD9 | 3804, 3814, 3834, 3922, 3924, 3925, 3954, 3978 |

Supplementary Table S2.Variance Inflation Factors (VIF) for Candidate Predictors

| Variable Name | VIF |
| --- | --- |
| Surgery Category | 1.10096676522287 |
| age | 1.18693392441423 |
| gender | 1.13782196426761 |
| race | 1.04034596331006 |
| sofa | 1.1703256806839 |
| Hypertension | 1.27732752284481 |
| Acute Kidney Injury | 1.1594421390291 |
| Liver Cirrhosis | 1.04047084280235 |
| Pneumonia | 1.05989616660727 |
| Cerebrovascular Accident | 1.02757968002311 |
| Chronic Kidney Disease | 1.32602577054024 |
| Cancer | 1.03677735166546 |
| Diabetes Mellitus | 1.19934798881814 |
| Heart Failure | 1.20960601861089 |
| Myocardial Infarction | 1.13201395476748 |
| Ischemic Heart Disease | 1.55226009924355 |
| COPD | 1.03473234925253 |
| CRRT | 1.19412003507248 |
| BMI | 1.11922881305134 |
| SBP | 1.22937579208182 |
| DBP | 1.24897143403846 |
| Respiratory Rate | 1.02970382330979 |
| SpO_2_ | 1.05789604293619 |
| Temperature | 1.03267199937985 |
| Anion Gap | 1.17242049888395 |
| Calcium Total | 1.05542467395191 |
| Potassium | 1.10252844602451 |
| Sodium | 1.08481587474111 |
| BUN | 1.27869652708602 |
| Hematocrit | 5.45850961751264 |
| Hemoglobin | 4.9973339484335 |
| INRPT | 7.4802863561523 |
| PLT | 1.1967333475088 |
| PT | 7.47078920542206 |
| RDW | 1.27820998541107 |
| RBC | 3.2124592844211 |
| WBC | 1.17775566325057 |
| Heart Rate | 1.07673177651499 |
| Ventilation hour | 1.20789710217678 |
| Statin | 1.01574592234051 |
| Amiodarone | 1.01974135709005 |
| Mg | 1.05318679530507 |
| Insulin | 1.04240439244742 |
| Vasopressor | 1.06332970752501 |
| Glucocorticoid | 1.01558124699062 |
| Beta-blocker | 1.03580090670176 |
| SHR | 1.1210619674432 |

Supplementary Table S3.Threshold Effect Analysis of HGI on POAF Using GLM

| Model Component | Odds Ratio (OR) | 95% CI | P-value |
| --- | --- | --- | --- |
| Model 1(Linear Effect) | 0.8965 | 0.8179 – 0.9795 | 0.0174 |
| Threshold Point (W) | 1.17 | – | – |
| Model 2: SHR < 1.17 | 0.8304 | 0.6944 – 0.9909 | 0.0404 |
| Model 2: SHR > 1.17 | 0.9451 | 0.8210 – 1.0783 | 0.4162 |
| Log-likelihood Ratio Test | – | – | 0.3261 |

Supplementary Table S4. Threshold Effect Analysis of SHR on POAF Using GLM

| Model Component | Odds Ratio (OR) | 95% CI | P-value |
| --- | --- | --- | --- |
| Model 1(Linear Effect) | 1.3865 | 1.0136 – 1.9306 | 0.0490 |
| Threshold Point (W) | 0.9067 | – | – |
| Model 2: SHR < 0.9067 | 3.1093 | 1.3477 – 7.2645 | 0.0082 |
| Model 2: SHR > 0.9067 | 1.0650 | 0.7495 – 1.5925 | 0.7387 |
| Log-likelihood Ratio Test | – | – | 0.0401 |

Supplementary Table S5. Threshold Effect Analysis of GV on POAF Using GLM

| Model Component | Odds Ratio (OR) | 95% CI | P-value |
| --- | --- | --- | --- |
| Model 1(Linear Effect) | 1.4378 | 0.6919 – 2.9836 | 0.3295 |
| Threshold Point (W) | 0.4714 | – | – |
| Model 2: SHR < 0.4714 | 0.7734 | 0.3047 – 1.9586 | 0.5881 |
| Model 2: SHR > 0.4714 | 17.0459 | 1.5356 – 228.7155 | 0.0245 |
| Log-likelihood Ratio Test | – | – | 0.0343 |

Supplementary Table S6. Association of Glycemic Variability within 3 Postoperative Days with POAF: Logistic Regression Analysis

| Outcomes exposure | Unjusted  OR (95% CI, P) | Model 1  OR (95% CI, P) | Model 2  OR (95% CI, P) | Model 3  OR (95% CI, P) | Model 4  OR (95% CI, P) |
| --- | --- | --- | --- | --- | --- |
| Q1 | Ref | Ref | Ref | Ref | Ref |
| Q2 | 1.05（0.85-1.30，P=0.6300） | 0.98 (0.78–1.22,P=0.8383) | 0.98 (0.78-1.22, P = 0.85) | 0.90 (0.71–1.13, P = 0.3569) | 0.86 (0.68-1.09,P=0.2203) |
| Q3 | 0.96（0.78-1.19，P=0.7066） | 0.87 (0.70–1.08, P = 0.2145) | 0.86 (0.69-1.08, P=0.1937) | 0.88 (0.69–1.12, P = 0.2952) | 0.89 (0.70-1.15,P=0.38) |
| P for trend | 0.98（0.88-1.09，P=0.7073） | 0.93(0.84-1.04，P=0.2139） | 0.93（0.83-1.04，P=0.1931） | 0.94（0.83-1.06，P=0.2908） | 0.94（0.83-1.07，P=0.3634） |
| Continuous | 1.25 (0.68-2.29, P=0.4809) | 1.01 (0.53-1.90, P=0.9823) | 1.00 (0.52-1.89, P=0.9915) | 1.14 (0.56-2.30, P=0.7248) | 1.24 (0.60-2.57, P=0.5594) |

Model 1 Age, Gender, Race, BMI

Model 2 +SBP, DBP, Respiratory Rate, SpO_2_, Heart rate、Temperature

Model 3 + Comorbidities (Hypertension、Acute Kidney Injury、Liver Cirrhosis、Pneumonia、Cerebrovascular Accident、Chronic Kidney Disease、Cancer、Diabetes Mellitus、Heart Failure、Myocardial Infarction、Ischemic Heart Disease、COPD) , Scores (SOFA), Surgery Category

Model 4 + Medications (Statin、Amiodarone、Mg、Vasopressor、Glucocorticoid、Beta-blocker、Insulin) + Lab Tests (anion gap、potassium、sodium、Calcium、BUN、hematocrit、Hemoglobin、INRPT、PLT、PT、RDW、RBC、ventilation hour、WBC) 、CRRT

Supplementary Figure S1. Restricted Cubic Spline Analysis of Glycemic Variability Within 72 Postoperative Hours and Risk of POAF


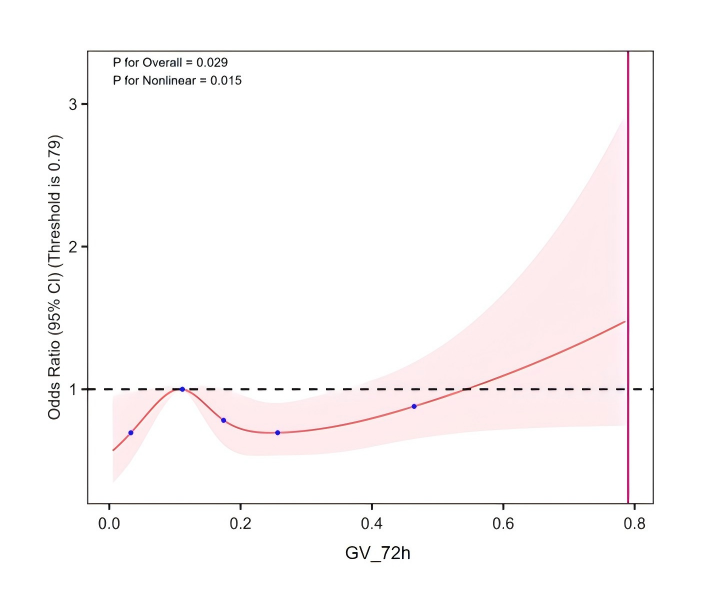


Supplementary Table S7. Threshold Effect of 3-Day Postoperative GV on POAF (GLM-Based Analysis)

| Model Component | Odds Ratio (OR) | 95% CI | P-value |
| --- | --- | --- | --- |
| Model 1(Linear Effect) | 1.2426 | 0.5977 – 2.5740 | 0.5594 |
| Threshold Point (W) | 0.4645 | – | – |
| Model 2: SHR < 0.4645 | 0.6182 | 0.2455 – 1.5510 | 0.3062 |
| Model 2: SHR > 0.4645 | 21.7587.0459 | 1.9313 – 311.9204 | 0.0160 |
| Log-likelihood Ratio Test | – | – | 0.0146 |

Supplementary Table S8. Association of Glycemic Variability with POAF in Patients with ≥3 Postoperative Glucose Measurements: A Logistic Regression Analysis

| Outcomes exposure | Unjusted  OR (95% CI, P) | Model 1  OR (95% CI, P) | Model 2  OR (95% CI, P) | Model 3  OR (95% CI, P) | Model 4  OR (95% CI, P) |
| --- | --- | --- | --- | --- | --- |
| Q1 | Ref | Ref | Ref | Ref | Ref |
| Q2 | 1.05（0.81-1.36，P=0.7140） | 0.98 (0.74–1.28,P=0.8620) | 0.97(0.74-1.28, P = 0.8525) | 0.88(0.66–1.17, P = 0.3650) | 0.84(0.62-1.12,P=0.2292) |
| Q3 | 1.00（0.77-1.30，P=0.9787） | 0.94 (0.71–1.24, P = 0.6751) | 0.94 (0.71-1.24, P=0.6420) | 0.90(0.66–1.23, P = 0.5207) | 0.90（0.65-1.25,P=0.5291) |
| P for trend | 1.00（0.87-1.14，P=0.9514） | 0.97（0.85-1.11，P=0.6717） | 0.97（0.84-1.11，P=0.6379） | 0.95（0.81-1.11，P=0.5277） | 0.95（0.81-1.12，P=0.5307） |
| Continuous | 1.38 (0.63-3.01, P=0.4159) | 1.38 (0.60-3.12, P=0.4445) | 1.37 (0.60-3.12, P=0.4549) | 1.49 (0.60-3.72, P=0.3909) | 1.50 (0.58-3.88, P=0.4004) |

Model 1 Age, Gender, Race, BMI

Model 2 +SBP, DBP, Respiratory Rate, SpO_2_, Heart rate、Temperature

Model 3 + Comorbidities (Hypertension、Acute Kidney Injury、Liver Cirrhosis、Pneumonia、Cerebrovascular Accident、Chronic Kidney Disease、Cancer、Diabetes Mellitus、Heart Failure、Myocardial Infarction、Ischemic Heart Disease、COPD) , Scores (SOFA), Surgery Category

Model 4 + Medications (Statin、Amiodarone、Mg、Vasopressor、Glucocorticoid、Beta-blocker、Insulin) + Lab Tests (anion gap、potassium、sodium、Calcium、BUN、hematocrit、Hemoglobin、INRPT、PLT、PT、RDW、RBC、ventilation hour、WBC) 、CRRT

Supplementary Figure S2. Restricted Cubic Spline Analysis of Glycemic Variability and POAF Based on ≥3 Postoperative Glucose Measurements


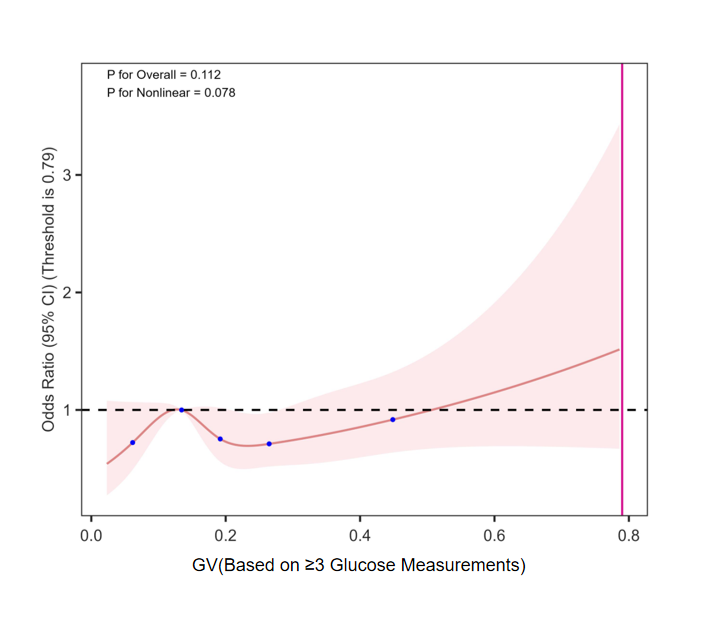


Supplementary Table S9. Training Set and Validation Set

| Variables | Total(n=2177) | Train(n=1741) | Test(n=436) | Statistic | P |
| --- | --- | --- | --- | --- | --- |
| Surgery_Category = CABG | 1082 (49.70%) | 888 (51.01%) | 194 (44.50%) | Fisher | 0.06990 |
| Surgery_Category = Valve | 613 (28.16%) | 468 (26.88%) | 145 (33.26%) | Fisher | 0.06990 |
| Surgery_Category = Aortic | 7 (0.32%) | 4 (0.23%) | 3 (0.69%) | Fisher | 0.06990 |
| Surgery_Category = CABG+Valve | 430 (19.75%) | 345 (19.82%) | 85 (19.50%) | Fisher | 0.06990 |
| Surgery_Category = CABG+Aortic | 3 (0.14%) | 3 (0.17%) | 0 (0.00%) | Fisher | 0.06990 |
| Surgery_Category = Valve+Aortic | 37 (1.70%) | 29 (1.67%) | 8 (1.83%) | Fisher | 0.06990 |
| Surgery_Category = CABG+Valve+Aortic | 5 (0.23%) | 4 (0.23%) | 1 (0.23%) | Fisher | 0.06990 |
| age | 69.00 (60.00, 76.00) | 69.00 (60.00, 76.00) | 68.00 (59.00, 76.00) | Z=1.14 | 0.25200 |
| sapsii | 35.00 (29.00, 43.00) | 36.00 (29.00, 43.00) | 35.00 (27.00, 42.00) | Z=1.51 | 0.13000 |
| charlson | 4.00 (3.00, 6.00) | 4.00 (3.00, 6.00) | 4.00 (3.00, 6.00) | Z=1.17 | 0.24100 |
| aki = 0 | 1648 (75.70%) | 1312 (75.36%) | 336 (77.06%) | χ²=0.46 | 0.49700 |
| aki = 1 | 529 (24.30%) | 429 (24.64%) | 100 (22.94%) | χ²=0.46 | 0.49700 |
| SHR | 0.96 (0.77, 1.15) | 0.96 (0.77, 1.15) | 0.97 (0.78, 1.17) | Z=0.72 | 0.47400 |
| Urea_Nitrogen | 16.00 (12.00, 21.00) | 16.00 (13.00, 22.00) | 15.00 (12.00, 20.00) | Z=2.65 | 0.00811 |
| INRPT | 1.40 (1.30, 1.60) | 1.50 (1.30, 1.60) | 1.40 (1.30, 1.60) | Z=0.88 | 0.37700 |
| PT | 15.80 (14.40, 17.50) | 15.90 (14.40, 17.50) | 15.65 (14.47, 17.50) | Z=0.75 | 0.45400 |
| RDW | 13.60 (12.90, 14.70) | 13.70 (13.00, 14.70) | 13.50 (12.88, 14.60) | Z=1.71 | 0.08800 |
| ventilation_hour | 38.73 (18.52, 64.00) | 39.00 (18.00, 64.80) | 37.86 (20.08, 61.22) | Z=0.12 | 0.90600 |
| POAF = 0 | 1310 (60.17%) | 1048 (60.20%) | 262 (60.09%) | χ²=0.00 | 1.00000 |
| POAF = 1 | 867 (39.83%) | 693 (39.80%) | 174 (39.91%) | χ²=0.00 | 1.00000 |

Supplementary Table S10. The performance comparison of each ML model in predicting POAF

|  | Sensitivity | Precision | F1 | Specificity | Accuracy | AUC |
| --- | --- | --- | --- | --- | --- | --- |
| GBM | 0.82 | 0.52 | 0.64 | 0.50 | 0.63 | 0.72 |
| LDA | 0.85 | 0.51 | 0.64 | 0.45 | 0.61 | 0.73 |
| Naive Bayes | 0.91 | 0.49 | 0.63 | 0.36 | 0.58 | 0.71 |
| MLP | 0.86 | 0.50 | 0.63 | 0.44 | 0.60 | 0.69 |
| Ridge | 0.85 | 0.50 | 0.63 | 0.44 | 0.61 | 0.72 |
| SVM | 0.83 | 0.51 | 0.63 | 0.47 | 0.61 | 0.71 |
| LogReg | 0.85 | 0.50 | 0.63 | 0.44 | 0.60 | 0.73 |
| AdaBoost | 0.85 | 0.50 | 0.63 | 0.43 | 0.60 | 0.74 |
| RandomForest | 0.91 | 0.46 | 0.61 | 0.30 | 0.54 | 0.70 |
| KNN | 0.91 | 0.46 | 0.61 | 0.30 | 0.54 | 0.68 |
| XGBoost | 0.95 | 0.44 | 0.60 | 0.19 | 0.50 | 0.67 |
| NN | 0.53 | 0.48 | 0.51 | 0.62 | 0.58 | 0.63 |
| DecisionTree | 0.53 | 0.47 | 0.50 | 0.61 | 0.58 | 0.57 |

Supplementary Figure S3. SHAP Summary Plot of the Initial AdaBoost Model

**
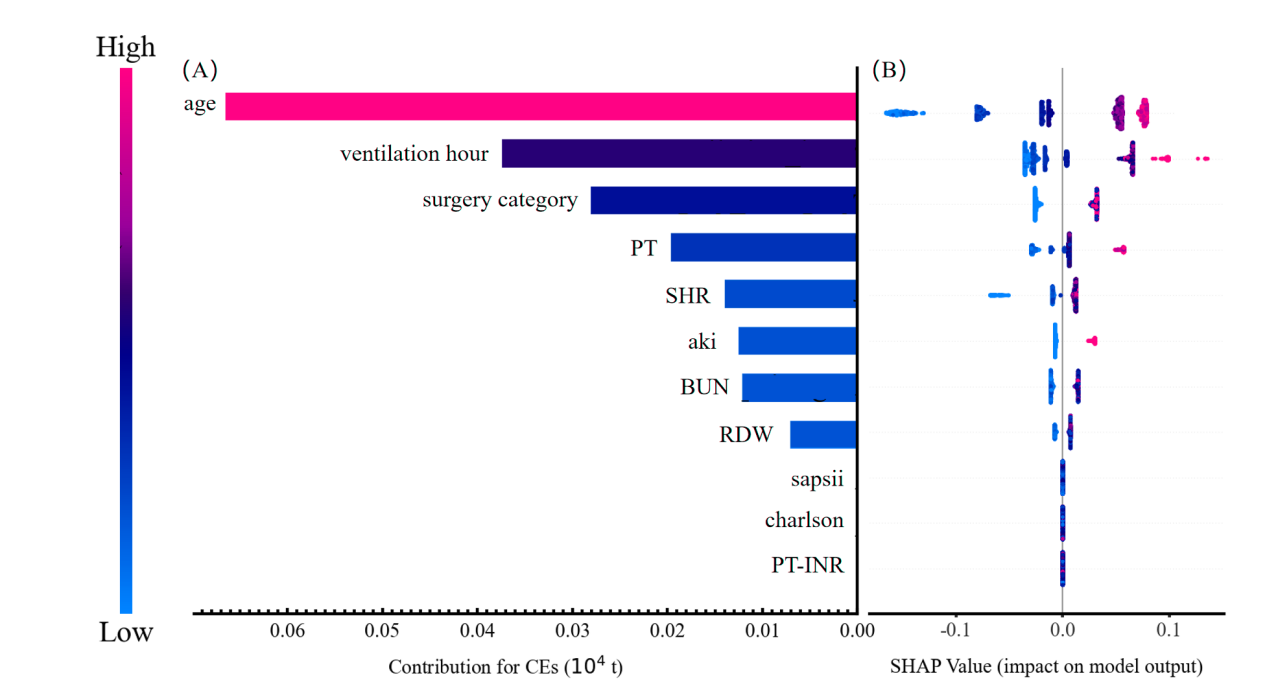
**

Supplementary Table S11. The performance comparison of Adaboost model in predicting POAF

|  | Recall(Sensitivity) | Precision | F1 | Specificity | Accuracy | ROC_AUC |
| --- | --- | --- | --- | --- | --- | --- |
| Adaboost | 0.68 | 0.68 | 0.65 | 0.88 | 0.68 | 0.74 |

Supplementary Figure S4. Screenshots of the POAF Risk Prediction System Website


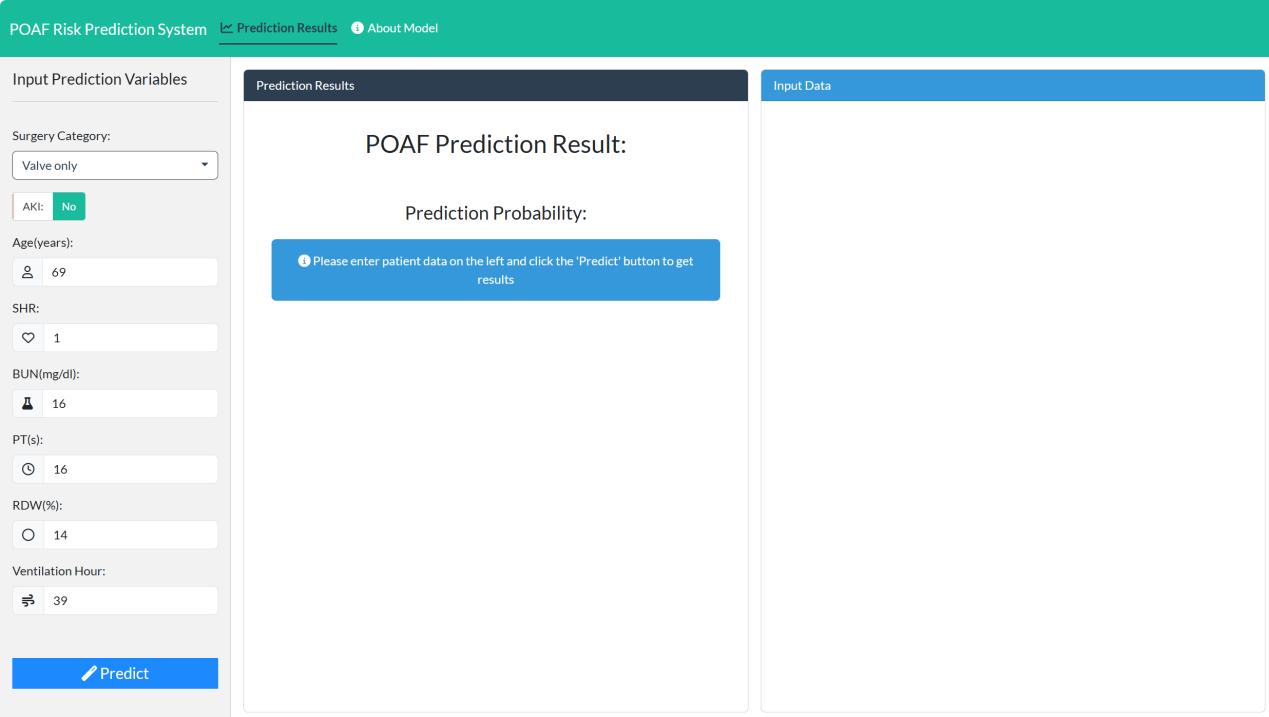


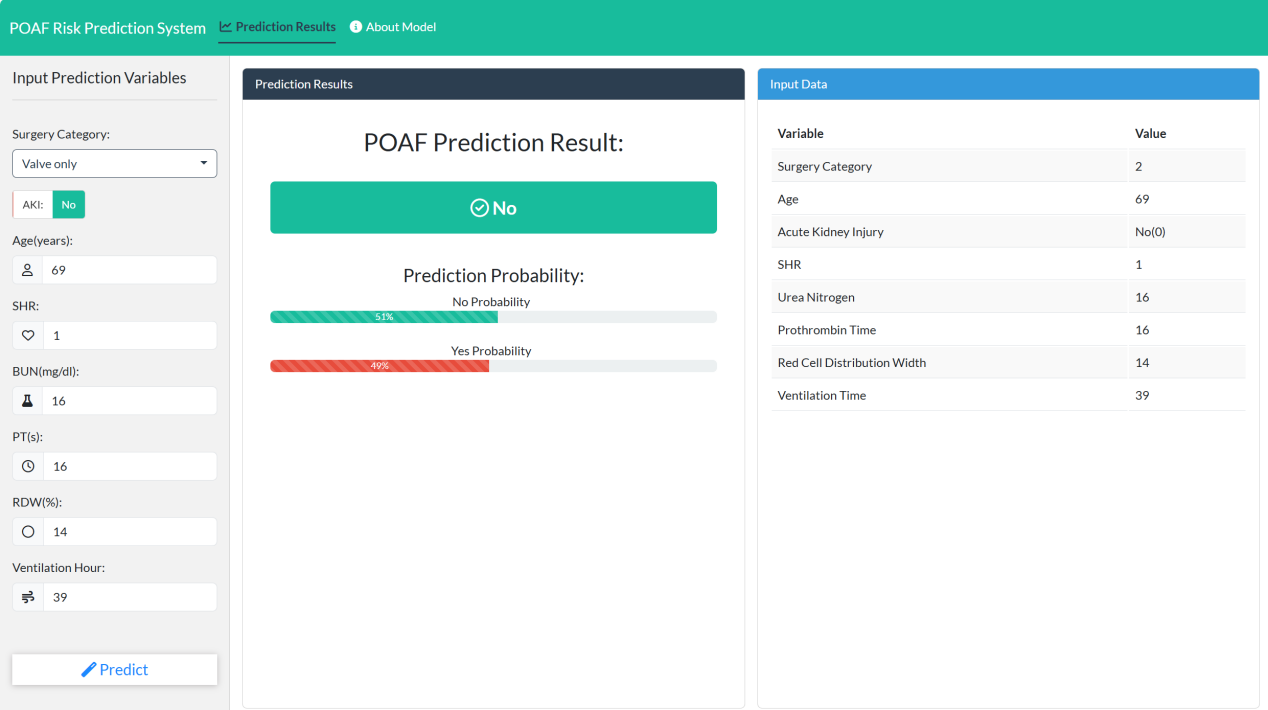

Supplement: Supplementary file 1 — Supplementary Material 1 [file 12911_2026_3507_MOESM1_ESM.docx]
